# Resolvin D1 engages FPR2–ARRB2 signaling and chromatin–microRNA regulation to restrain inflammatory activation in bovine mammary epithelial cells

**DOI:** 10.3389/fimmu.2026.1978720

**Published:** 2026-10-23

**Authors:** Naihan Yuan, Yanfei Feng, Zhuo Li, Pengfei Lin, Aihua Wang, Yaping Jin

**Affiliations:** 1 College of Veterinary Medicine, Northwest A&F University, Yangling, China; 2 Key Laboratory of Animal Biotechnology, Ministry of Agriculture and Rural Affairs, Northwest A&F University, Yangling, China

**Keywords:** bovine mastitis, chromatin regulation, inflammatory response, mammary epithelial cell, microRNA, resolution

## Abstract

**Introduction:**

Mammary epithelial cells are active participants in local innate immunity, mounting NF-κB-dependent cytokine responses to inflammatory stimuli. Excessive or persistent activation of this pathway, however, can sustain inflammation. Resolvin D1 (RvD1) is a lipid mediator with pro-resolving activity, but the specific mechanism by which RvD1 regulates inflammation in bovine mammary epithelial cells remains to be elucidated.

**Methods:**

Using an LPS-induced inflammatory model in MAC-T bovine mammary epithelial cells, with selected responses confirmed at predefined time points in primary bovine mammary epithelial cells, this study investigated how RvD1 pretreatment attenuates subsequent LPS-induced inflammatory activation.

**Results:**

The results showed that RvD1 significantly inhibited LPS-induced mammary epithelial inflammation, and that its early anti-inflammatory effect involved FPR2/ARRB2-associated suppression of NF-κB signaling. Further investigation showed that ARRB2 contributed to the RvD1-mediated reduction in p65 and HDAC1 enrichment at the MEG3 promoter-proximal region and the restoration of H3K27ac at this region. Meanwhile, RvD1 increased CREB phosphorylation in an FPR2-sensitive manner and increased CREB enrichment at this region; CREB1 knockdown attenuated the RvD1-associated increases in MEG3 and DLK1–DIO3 locus-associated miRNAs. Among these miRNAs, bta-miR-380-5p and bta-miR-654 targeted TAB2 and IL-33, respectively, and contributed to the later suppression of IL-6 and IL-8 secretion by RvD1.

**Discussion:**

These findings identify receptor-proximal, chromatin, and microRNA-associated mechanisms underlying the anti-inflammatory response to RvD1 in bovine mammary epithelial cells.

## Introduction

Mastitis is one of the most common and economically damaging diseases in dairy cattle (1, 2). Mammary epithelial cells not only constitute the local mammary barrier but also actively participate in local innate immune responses to inflammatory stimulation (3). During bovine mastitis, activation of inflammatory signaling in mammary epithelial cells promotes cytokine and chemokine production and contributes to the amplification of local inflammation, whereas excessive or prolonged inflammatory responses can aggravate mammary tissue damage and impair lactation (4). However, antimicrobial resistance and concerns regarding antibiotic residues in milk complicate mastitis control and raise food-safety concerns (2, 5). Therefore, in addition to controlling pathogen infection, limiting the sustained amplification of local mammary inflammation and promoting inflammation resolution are important for restoring mammary immune homeostasis during bovine mastitis.

Resolution of inflammation is not a passive attenuation of inflammatory signaling but a biological process actively regulated by endogenous mediators (6). Specialized pro-resolving mediators are endogenous lipid mediators derived from polyunsaturated fatty acids. During the later stages of inflammation, they can promote the cessation of inflammatory-cell infiltration, enhance the clearance of apoptotic cells and cellular debris, and facilitate the return of the local inflammatory microenvironment to homeostasis (7, 8). Resolvin D1 (RvD1) is a representative docosahexaenoic acid-derived specialized pro-resolving mediator that can limit excessive inflammation and promote inflammation resolution through receptors including lipoxin A4 receptor/formyl peptide receptor 2 (ALX/FPR2) and G protein-coupled receptor 32 (GPR32) (9). The internalization and trafficking of ALX/FPR2, a G protein-coupled receptor, involve β-arrestin (10, 11), and β-arrestin 2 (ARRB2) has been reported to interact with inhibitor of nuclear factor κB alpha (IκBα) and limit nuclear factor κB (NF-κB) activation (12). However, whether RvD1 engages ARRB2 downstream of FPR2 to restrain NF-κB signaling in bovine mammary epithelial cells has not been established.

Inflammatory responses are regulated not only by receptor-proximal signaling and transcriptional control, but also by post-transcriptional mechanisms that influence the stability and translation of inflammatory mRNAs (13). MicroRNAs (miRNAs) are small non-coding RNAs approximately 18–25 nt in length that can bind to the 3′ untranslated region (UTR) of target mRNAs to inhibit translation or promote mRNA degradation, thereby regulating multiple inflammation-related genes (14, 15). RvD1 can engage this regulatory layer by altering miRNA expression. In a self-limited acute inflammation model, RvD1 induced a receptor-dependent pro-resolving miRNA expression program (16). RvD1 has also been reported to induce distinct miRNA signatures and to regulate miR-21 and miR-155 together with Toll-like receptor-related inflammatory genes (17, 18). Importantly, miRNAs can actively participate in pro-resolving responses rather than simply reflect changes in inflammatory activity. For example, miR-466l promotes the biosynthesis of specialized pro-resolving mediators, including RvD1 and RvD5, and accelerates inflammatory resolution, whereas RvD1 in turn reduces miR-466l expression in human macrophages (19). miRNA genes may occur independently or in clusters and may be co-transcribed as polycistronic transcripts, allowing miRNAs within a cluster to coordinately regulate multiple components of the same signaling pathway (20). The delta-like noncanonical Notch ligand 1–iodothyronine deiodinase 3 (DLK1–DIO3) imprinted region contains the maternally expressed 3 (MEG3) long noncoding RNA (lncRNA), small nucleolar RNAs, and a cluster of miRNAs (21). The maternally expressed non-coding RNA domain includes MEG3 and a large downstream miRNA cluster, and the epigenetic state of the MEG3 promoter-proximal region is associated with the transcriptional activity of this domain (22, 23). miRNAs derived from this region participate in development, metabolism, and cell-fate regulation (24), and altered expression of these miRNAs has also been observed in immune-mediated and inflammatory diseases (25). Together, these studies indicate that RvD1 can act at both receptor-proximal and post-transcriptional regulatory levels. However, whether these regulatory layers are linked to subsequent chromatin regulation and DLK1–DIO3-associated miRNA expression in bovine mammary epithelial cells remains unclear.

Because LPS is a well-characterized TLR4 agonist that activates NF-κB signaling and inflammatory cytokine responses in bovine mammary epithelial cells (26), we used an LPS-induced inflammatory model with RvD1 pretreatment to examine whether RvD1 acts through FPR2–ARRB2 signaling to attenuate subsequent inflammatory activation and how this early signaling is connected to subsequent chromatin regulation and DLK1–DIO3 miRNA expression. We hypothesized that RvD1 pretreatment attenuates LPS-induced inflammatory responses in bovine mammary epithelial cells through coordinated receptor-proximal, chromatin, and miRNA-mediated mechanisms.

## Materials and methods

### Cell culture and treatments

MAC-T cells, an immortalized bovine mammary epithelial cell line maintained in our laboratory, were cultured in DMEM/F12 medium (SH30023.01, Cytiva, Logan, UT, USA) supplemented with 10% FBS (Excellent-FBS, Cat. No. BE500, Newzerum Ltd., Shanghai, China) and maintained in a humidified incubator at 37 °C with 5% CO_2_. The culture medium was replaced every 2 d. Cells were passaged when they reached approximately 80% confluence and seeded into culture plates for subsequent experiments. For cryopreservation, cells were resuspended in cell freezing medium (AR0003-100, Milecell Biological Science & Technology Co., Ltd., Shanghai, China) and stored under cryogenic conditions. Primary bovine mammary epithelial cells were previously established in our laboratory from mammary tissues collected from three lactating cows after routine slaughter at a local abattoir using a tissue-explant outgrowth method and were subsequently cryopreserved. Detailed lactation-stage information, including days in milk and classification as early, mid, or late lactation, was not recorded when the cells were originally established. Mammary tissues were rinsed with 75% ethanol, washed with sterile PBS, cut into approximately 1 mm³ fragments, and placed in culture dishes for explant outgrowth. Epithelial-like cells migrating from the tissue fragments were expanded and subsequently cryopreserved. The cryopreserved cells were recovered and cultured in DMEM/F12 medium supplemented with 10% FBS under the same conditions as MAC-T cells. Cells from the three donors were maintained separately, and cells at passage 5 or earlier were used. For validation experiments, primary cells were seeded in 6-well plates at 5 × 10^5^ cells/well and incubated overnight before treatment. For both MAC-T and primary bovine mammary epithelial cells, LPS (HY-D1056, MedChemExpress, Monmouth Junction, NJ, USA) was used at a final concentration of 1 μg/mL to induce an inflammatory response. This concentration was selected based on previous studies in MAC-T bovine mammary epithelial cells that used 1 μg/mL LPS to induce inflammatory responses (27, 28). For validation of LPS-responsive signaling in MAC-T cells, cells were stimulated with LPS (1 μg/mL) for 0, 15, 30, 60, or 120 min to assess IRF3 phosphorylation and TRAM–TRIF association. TLR4 protein expression was assessed in untreated control cells and cells stimulated with LPS (1 μg/mL) for 1 h. RvD1 (R10436, Psaitong, Beijing, China) was added at a final concentration of 100 nM 1 h before LPS stimulation. Primary cells were assigned to control, LPS, RvD1, and LPS plus RvD1 groups. The pretreatment design was used to examine how RvD1-responsive signaling modulates the subsequent early inflammatory response to LPS. Cells were collected after 1 h of LPS stimulation for Western blot analysis of p-p65, p65, IκBα, and p-IκBα; after 12 h for RT-qPCR; and after 36 h for Western blot analysis of TAB2 and IL-33. Culture supernatants were collected after 48 h for ELISA. These time points were selected based on the corresponding experiments in MAC-T cells, including the kinetic responses where applicable, and were used to assess whether selected responses could be reproduced in primary bovine mammary epithelial cells. WRW4 (SJ-MX0720, SparkJade, Shandong, China), an FPR2 antagonist, was added at a final concentration of 10 μM 1 h before RvD1 treatment. Forskolin (F127328, Aladdin, Shanghai, China) was used at 10 μM for 30 min as a positive control for CREB activation. Cells were collected at the indicated time points for subsequent analyses.

Cell transfection was performed using TurboFect Transfection Reagent (R0533, Thermo Fisher Scientific, Waltham, MA, USA) and Opti-MEM (31985070, Gibco, Thermo Fisher Scientific, Waltham, MA, USA) according to the manufacturers’ instructions. siRNAs, miRNA mimics, miRNA inhibitors, dual-luciferase reporter plasmids, target-expression plasmids, and their corresponding negative controls were transfected into cells for 48 h before drug treatment or subsequent analysis. Three siRNAs targeting ARRB2 were screened by Western blotting 48 h after transfection, and siARRB2-3, which produced the greatest reduction in ARRB2 protein abundance, was selected for subsequent experiments. Knockdown by siRNAs targeting RELA/p65, HDAC1, and CREB1 was also verified by Western blotting before functional analyses. To verify the effectiveness of miRNA modulation, miRNA overexpression following mimic transfection was confirmed by RT-qPCR, whereas the functional activity of the miRNA inhibitors was assessed by examining the derepression of the corresponding endogenous target proteins, TAB2 and IL-33, by Western blotting. For target-rescue experiments, bovine TAB2 and IL-33 expression constructs containing the complete coding sequence and corresponding 3′ UTR were used. The oeTAB2-WT and oeIL-33-WT constructs retained the native bta-miR-380-5p- and bta-miR-654-binding sites, respectively, whereas oeTAB2-MUT and oeIL-33-MUT carried nucleotide substitutions restricted to the corresponding miRNA seed-matching sites without altering the target-protein coding sequences. Cells were co-transfected with the indicated miRNA mimic and empty vector, WT target-expression plasmid, or MUT target-expression plasmid for 48 h. After LPS stimulation, cell lysates were collected at 36 h for Western blotting, and culture supernatants were collected at 48 h for ELISA. The siRNAs and their corresponding negative controls were synthesized by Sangon Biotech (Shanghai, China), whereas the miRNA mimics, miRNA inhibitors, dual-luciferase reporter plasmids, target-expression plasmids, and their corresponding negative controls were synthesized by General Biol Co., Ltd. (Anhui, China).

### Western blotting

Cells were lysed in RIPA buffer (PC101, Epizyme Biomedical Technology Co., Ltd., Shanghai, China) containing protease and phosphatase inhibitors (GRF103, Epizyme Biomedical Technology Co., Ltd., Shanghai, China). Total protein concentrations were determined using a BCA protein assay kit (ZJ101, Epizyme Biomedical Technology Co., Ltd., Shanghai, China). Equal amounts of protein were separated by SDS-PAGE and transferred onto PVDF membranes (0.2 μm; 03010040001, Sigma-Aldrich, St. Louis, MO, USA). After blocking with 5% non-fat milk or BSA, membranes were incubated overnight at 4 °C with primary antibodies against p-p65 (abs130624, Absin, Shanghai, China; 1:1000), p65 (A87175, Nature Biosciences, Hangzhou, China; 1:1000), IκBα (A12290, Nature Biosciences, Hangzhou, China; 1:1000), TLR4 (bs-20594R, Bioss, Beijing, China; 1:1000), TRIF (23288-1-AP, Proteintech, Wuhan, China; 1:1000), TRAM (sc-376076, Santa Cruz Biotechnology, Dallas, TX, USA; 1:1000), p-IRF3 (29528-1-AP, Proteintech, Wuhan, China; 1:1000), IRF3 (11312-1-AP, Proteintech, Wuhan, China; 1:1000), FPR2 (NLS1878, Novus Biologicals, Centennial, CO, USA; 1:1000), p-IκBα (82349-1-RR, Proteintech, Wuhan, China; 1:1000), p-IKK (A53955, Nature Biosciences, Hangzhou, China; 1:1000), IKK (A48244, Nature Biosciences, Hangzhou, China; 1:1000), p-TAK1 (A78585, Nature Biosciences, Hangzhou, China; 1:1000), TAK1 (abs124656, Absin, Shanghai, China; 1:1000), p-p38 (28796-1-AP, Proteintech, Wuhan, China; 1:1000), p38 (RM8093, Biodragon, Suzhou, China; 1:1000), TAB2 (82948-5-RR, Proteintech, Wuhan, China; 1:1000), IL-33 (A11378, Nature Biosciences, Hangzhou, China; 1:1000), p-CREB (abs154911, Absin, Shanghai, China; 1:1000), CREB (abs173590; Absin, Shanghai, China; 1:1000), ARRB2 (A63739, Nature Biosciences, Hangzhou, China; 1:1000), HDAC1 (A65412, Nature Biosciences, Hangzhou, China; 1:1000), Lamin B1 (A52835, Nature Biosciences, Hangzhou, China; 1:1000), GAPDH (A10139, Nature Biosciences, Hangzhou, China; 1:1000), and β-actin (20536-1-AP, Proteintech, Wuhan, China; 1:1000). Membranes were then incubated with Multi-rAb HRP-conjugated goat anti-rabbit IgG (H+L) (RGAR001, Proteintech, Wuhan, China) or HRP-conjugated goat anti-mouse IgG (H+L) (SA00001-1, Proteintech, Wuhan, China), according to the host species of the primary antibody. Immunoreactive bands were visualized using enhanced chemiluminescence reagent (IM101, YoungGen, Kunming, China) and imaged using an Amersham ImageQuant 800 imaging system (Cytiva, Marlborough, MA, USA).

Band intensities were quantified using ImageJ software. β-actin was used as the loading control for total protein samples. Lamin B1 and GAPDH were used as nuclear and cytoplasmic loading controls, respectively. p-p65, p-IKK, p-TAK1, p-p38, p-CREB, and p-IRF3 were normalized to their corresponding total proteins, whereas IκBα and p-IκBα were normalized to β-actin. Other target proteins, including TLR4, were normalized to the corresponding loading controls indicated in each figure.

### Nuclear and cytoplasmic protein extraction

Nuclear and cytoplasmic proteins were extracted using a nuclear and cytoplasmic protein extraction kit (BB-36021, Bestbio, Shanghai, China) according to the manufacturer’s instructions. The nuclear fraction was used to detect p65 nuclear translocation by Western blot. Lamin B1 and GAPDH were used as nuclear and cytoplasmic loading controls, respectively.

### Quantitative real-time PCR

Total RNA was extracted using AG RNAex Pro Reagent (AG21101, Accurate Biotechnology (Hunan) Co., Ltd., Changsha, China) according to the manufacturer’s protocol. For mRNA and lncRNA detection, RNA was reverse-transcribed into cDNA using a reverse transcription kit (MR201, Vazyme Biotech Co., Ltd., Nanjing, China).

For miRNA detection, genomic DNA was first removed using the miRNA 1st Strand cDNA Synthesis Kit (by stem-loop) (MR101, Vazyme Biotech Co., Ltd., Nanjing, China). Briefly, each 10-μL genomic DNA removal reaction contained 2 μL of 5× gDNA Wiper Mix, 10 pg–1 μg total RNA, and RNase-free water, and was incubated at 42 °C for 2 min. The 20-μL reverse-transcription reaction contained 10 μL of the genomic DNA removal reaction, 1 μL of 2 μM stem-loop primer, 2 μL of 10× RT Mix, 2 μL of HiScript II Enzyme Mix, and RNase-free water. Reverse transcription was performed at 25 °C for 5 min, 50 °C for 15 min, and 85 °C for 5 min. The stem-loop RT primer sequences are provided in **Supplementary Table S2A**. qPCR was performed using SYBR Green Premix Pro Taq HS qPCR Kit (AG11701, Accurate Biotechnology (Hunan) Co., Ltd., Changsha, China) on a QuantStudio 6 Flex Real-Time PCR System (Applied Biosystems, Foster City, CA, USA). Each 20-μL qPCR reaction contained 10 μL of 2× SYBR Green Pro Taq HS Premix, less than 100 ng of cDNA template, 0.4 μL each of 10 μM forward and reverse primers, 0.4 μL of 4 μM ROX Reference Dye, and RNase-free water. Amplification was performed at 95 °C for 30 s, followed by 40 cycles of 95 °C for 5 s and 60 °C for 30 s, with a subsequent dissociation stage. Primer sequences used for mRNA and lncRNA detection and miRNA detection are provided in **Supplementary Tables S2B** and **S2C**. All primers were synthesized by Sangon Biotech (Shanghai, China). Primer specificity and amplification efficiency were experimentally validated, and melting-curve analysis confirmed the generation of single specific amplification products. For both MAC-T and primary bovine mammary epithelial cells, *GAPDH* was used as the internal control for mRNA and lncRNA detection, whereas bta-miR-16a was used as the internal control for miRNA detection. Relative expression levels were calculated using the 2^−ΔΔCt^ method.

### ELISA

Cell culture supernatants were collected after treatment and centrifuged to remove cell debris. For MAC-T cells, IL-6 and IL-8 concentrations were measured using commercial bovine ELISA kits (IL-6, JL22488; IL-8, JL22489; JONLNBIO, Shanghai, China) according to the manufacturer’s instructions. For primary bovine mammary epithelial cells, IL-6 and IL-8 concentrations were measured using the same bovine IL-6 and IL-8 ELISA kits described above. Absorbance was measured at 450 nm using a Spark multimode microplate reader (Tecan, Männedorf, Switzerland). Cytokine concentrations were calculated from the corresponding standard curves.

### Immunofluorescence staining

Cells were seeded on glass coverslips. Where indicated, cells were pretreated with WRW4 for 1 h and subsequently treated with RvD1 for 40 min. After treatment, cells were fixed with 4% paraformaldehyde for 15 min at room temperature, permeabilized with 0.1% Triton X-100 for 5 min at room temperature, and blocked with BSA for 30 min at room temperature. Sequential TSA-based immunofluorescence staining was performed. Cells were first incubated overnight at 4 °C with the primary antibody against FPR2 (NLS1878, Novus Biologicals, Centennial, CO, USA; 1:50), followed by incubation with an HRP-conjugated anti-rabbit secondary antibody (RGAU011, Proteintech, Wuhan, China) for 30 min at room temperature. The FPR2 signal was developed using CoraLite Plus 488-Tyramide Reagent (PR30022, Proteintech, Wuhan, China) for 10 min at room temperature in the dark. After antibody stripping and inactivation of residual HRP activity, cells were incubated overnight at 4 °C with the primary antibody against ARRB2 (A63739, Nature Biosciences, Hangzhou, China; 1:50), followed by incubation with the same HRP-conjugated anti-rabbit secondary antibody for 30 min at room temperature. The ARRB2 signal was developed using CoraLite Plus 594-Tyramide Reagent (PR30024, Proteintech, Wuhan, China) for 10 min at room temperature in the dark. Nuclei were counterstained with DAPI staining solution (PR30021, Proteintech, Wuhan, China) for 10 min at room temperature in the dark. Images were acquired using a ZEISS Axio Imager. M2 fluorescence microscope (Carl Zeiss, Oberkochen, Germany) under identical acquisition settings across the experimental groups. FPR2–ARRB2 colocalization was quantified using Pearson’s correlation coefficient calculated in ImageJ. Data were obtained from three independent experiments.

### Co-immunoprecipitation

Cells were lysed in IP lysis buffer (PC105, Epizyme Biomedical Technology Co., Ltd., Shanghai, China) supplemented with protease and phosphatase inhibitors. For the ubiquitination assay, the lysis buffer was additionally supplemented with 10 mM N-ethylmaleimide (N755648, Aladdin, Shanghai, China) to inhibit deubiquitinating activity and preserve protein ubiquitination. After centrifugation, 5% of each cell lysate was retained as the input fraction. The remaining lysates were incubated overnight at 4 °C with 2 μg of the indicated IP antibody against ARRB2, IκBα, p65, or TRAM (sc-376076, Santa Cruz Biotechnology, Dallas, TX, USA). An equal amount of normal rabbit IgG (2 μg; 30000-0-AP, Proteintech, Wuhan, China) or normal mouse IgG (2 μg; B900620, Proteintech, Wuhan, China) was used as the negative control, according to the host species of the IP antibody. Immunocomplexes were captured using a Protein A+G Magnetic Bead Immunoprecipitation Kit (P2180S, Beyotime, Shanghai, China), with magnetic beads added according to the manufacturer’s recommendations. The immunoprecipitated complexes were washed, eluted, and analyzed by Western blotting. For Western blot detection of immunoprecipitated samples, HRP-conjugated anti-rabbit IgG for IP (RA1008-01, Vazyme Biotech Co., Ltd., Nanjing, China) or anti-mouse IgG for IP (RA1009-01, Vazyme Biotech Co., Ltd., Nanjing, China) was used according to the host species of the primary antibody.

Five IP-based analyses were performed. First, ARRB2 was immunoprecipitated, and the precipitated complexes were immunoblotted for FPR2 to examine the association between ARRB2 and FPR2. Second, ARRB2 was immunoprecipitated, and the precipitated complexes were immunoblotted for IκBα to examine the association between ARRB2 and IκBα. Third, IκBα was immunoprecipitated, and its K48-linked polyubiquitination was detected using an anti-K48-linked ubiquitin antibody (AWA00827, Abiowell, Changsha, China). For this ubiquitination assay, cells were treated with MG132 (10 μM; HY-13259, MedChemExpress, Monmouth Junction, NJ, USA) beginning 2 h before RvD1 pretreatment. MG132 was maintained in the culture medium throughout the subsequent RvD1 and LPS treatments, resulting in a total exposure time of 4 h. Fourth, nuclear extracts were subjected to immunoprecipitation with an anti-p65 antibody, and the precipitated complexes were immunoblotted for HDAC1 to examine the nuclear association between p65 and HDAC1. Fifth, TRAM was immunoprecipitated from cells stimulated with LPS for 0, 15, 30, 60, or 120 min, and the precipitated complexes were immunoblotted for TRIF to examine the time-dependent association between TRAM and TRIF.

### CUT&Tag-qPCR

CUT&Tag was performed using 1 × 10^5^ cells per reaction with the AccuNext CUT&Tag DNA Library Prep Kit for Illumina (AG12555, Accurate Biotechnology (Hunan) Co., Ltd., Changsha, China) according to the manufacturer’s instructions. Briefly, cells were immobilized on concanavalin A-coated magnetic beads, permeabilized, and incubated with primary antibodies against p65 (A87175, Nature Biosciences, Hangzhou, China), HDAC1 (A65412, Nature Biosciences, Hangzhou, China), H3K27ac (82902-1-RR, Proteintech, Wuhan, China), or CREB (abs173590, Absin, Shanghai, China). Normal rabbit IgG (30000-0-AP, Proteintech, Wuhan, China) was used as the negative control. After incubation with the corresponding secondary antibodies, antibody-bound chromatin was fragmented using protein A/G–Tn5 transposase. The fragmented DNA was then extracted, purified, and directly subjected to qPCR analysis. CUT&Tag-qPCR experiments were performed in three independent biological replicates.

For p65 and HDAC1 CUT&Tag-qPCR, cells were collected 1 h after LPS stimulation. For H3K27ac CUT&Tag-qPCR, cells were collected 8 h after LPS stimulation. For CREB1 CUT&Tag-qPCR, cells were transfected with siCREB or siNC for 48 h, pretreated with RvD1 where indicated for 1 h, and then stimulated with LPS for 1 h. The enrichment of p65 and HDAC1 at three predicted p65-binding regions within the *MEG3* promoter was assessed using three qPCR primer sets, designated p65-P1, p65-P2, and p65-P3. The same primer sets were used to assess H3K27ac enrichment at the corresponding *MEG3* promoter regions. Primer sequences and amplicon sizes are provided in **Supplementary Table S2D**.

CREB1 enrichment at the predicted CREB1-binding regions within the *MEG3* promoter was assessed using two qPCR primer sets, designated CREB1-P1 and CREB1-P2. CREB1-P1 encompassed the CREB1–1 binding site, whereas CREB1-P2 encompassed the adjacent CREB1–2 and CREB1–3 binding sites. Primer sequences and amplicon sizes are provided in **Supplementary Table S2D**. Enrichment was calculated according to the manufacturer’s instructions and expressed as fold enrichment relative to the corresponding IgG control.

### JASPAR and STRING prediction

Potential RELA/p65- and CREB1-binding sites within the region spanning −2000 to +500 bp relative to the *MEG3* transcription start site were predicted using the JASPAR CORE vertebrate profiles MA0107.1 for RELA and MA0018.5 for CREB1, respectively. Predicted sites with a relative score of ≥0.80 were retained. Candidate sites were selected according to their relative scores and positions relative to the transcription start site, and CUT&Tag-qPCR primers were designed to amplify the corresponding *MEG3* promoter-proximal regions.

Potential interactions between RELA/p65 and HDAC family members were predicted using the STRING database.

### Small RNA sequencing and differential miRNA analysis

Following the indicated treatments, three independent biological replicates were collected from each of the control, LPS-stimulated, and RvD1-pretreated LPS-stimulated groups and submitted to OmicsMaster Biotechnology Co., Ltd. (Guangzhou, China) for small RNA sequencing and bioinformatic analysis. Small RNA libraries were sequenced on the Illumina NovaSeq X platform using a single-end 50-bp strategy.

Raw reads were filtered to remove low-quality reads, adaptor sequences, reads containing excessive ambiguous bases, and reads of unsuitable lengths. Clean reads were mapped to the *Bos taurus* ARS-UCD2.0 reference genome (GCA_002263795.4) obtained from Ensembl release 115 using Bowtie (version 1.1.2). Known miRNAs were annotated against miRBase (version 22.1). Low-abundance miRNAs were filtered and TMM-normalized. Count data were analyzed for differential expression using edgeR (29). Differentially expressed miRNAs were defined using |log_2_FC| > 1 and FDR < 0.05, with FDR representing the P value adjusted for multiple testing. Comparison labels followed the convention used by the analysis platform, in which A-versus-B denotes expression in B relative to A; accordingly, log_2_FC was calculated as log_2_(B/A). Volcano plots, Venn diagrams, hierarchical clustering heatmaps, and correlation heatmaps were generated from the processed sequencing data.

### miRNA target prediction and functional enrichment analysis

Potential target genes of candidate *DLK1–DIO3* cluster miRNAs were primarily predicted using TargetScan (version 7.0), with miRanda (version 3.3a) used as a complementary prediction tool. Genes predicted by at least one of the two algorithms were retained for subsequent functional enrichment and regulatory network analyses. Functional enrichment of the predicted target genes was analyzed for Gene Ontology (GO) terms and KEGG pathways. GO annotations were obtained using GO.db (version 3.14.0), and KEGG pathway information was based on KEGG Release 101. Enrichment significance was assessed using a one-tailed hypergeometric test implemented with the phyper function in R (lower.tail = FALSE). P values were adjusted for multiple comparisons using the p.adjust function with method = “fdr”. miRNA–target gene regulatory networks were constructed using Cytoscape.

### Dual-luciferase reporter assay

The bovine TAB2 and *IL-33* 3′ UTR sequences were retrieved from the NCBI database. Wild-type 3′ UTR fragments containing the predicted binding sites for bta-miR-380-5p and bta-miR-654 were amplified and cloned into the pmirGLO dual-luciferase reporter vector. Mutant reporter vectors were generated by mutating the corresponding miRNA seed-matching sequences within the predicted binding sites.

Bovine mammary epithelial cells were co-transfected with bta-miR-380-5p mimic or mimic NC and TAB2-WT or TAB2-MUT reporter plasmids. Similarly, cells were co-transfected with bta-miR-654 mimic or mimic NC and IL-33-WT or IL-33-MUT reporter plasmids. Transfection was performed using TurboFect Transfection Reagent (Thermo Fisher Scientific, Waltham, MA, USA) according to the manufacturer’s instructions.

After 48 h of transfection, firefly and Renilla luciferase activities were measured using a Dual Luciferase Reporter Gene Assay Kit (MH107, Coolaber, Beijing, China) on a Spark multimode microplate reader (Tecan, Männedorf, Switzerland). Relative luciferase activity was calculated by normalizing firefly luciferase activity to Renilla luciferase activity.

### Statistical analysis

All experiments were independently performed three times. No formal sample-size calculation or randomization was used. Blinding was not used because outcomes were measured using standardized assays. No samples were excluded. The experimental unit was an independently cultured and treated cell sample. Technical replicate wells were averaged within each experiment before statistical analysis. Data are presented as the mean ± SEM. Statistical analyses were performed using GraphPad Prism. Comparisons between two groups were performed using a two-tailed Student’s t-test. Comparisons among selected treatment groups were performed using one-way ANOVA followed by Šídák’s test for prespecified comparisons. Single-factor time-course experiments were analyzed using one-way ANOVA followed by Šídák’s test for prespecified comparisons, whereas complete two-factor designs were analyzed using two-way ANOVA followed by Šídák’s test for prespecified simple-effects comparisons. P < 0.05 was considered statistically significant. ns, not significant; *P < 0.05; **P < 0.01.

## Results

### RvD1 attenuates LPS-induced inflammatory activation in bovine mammary epithelial cells

Before evaluating the effects of RvD1, we first verified the responsiveness of the LPS–TLR4 signaling system in MAC-T cells. TLR4 protein was readily detectable under basal and LPS-stimulated conditions. LPS stimulation increased TRAM–TRIF association and IRF3 phosphorylation over time, with both reaching their highest observed levels at 60 min, supporting activation of the MyD88-independent TLR4 signaling branch (**Supplementary Figure S1**). To determine the inhibitory effect of RvD1 on mammary epithelial inflammation, cells were pretreated with RvD1 before LPS stimulation. Compared with LPS alone, RvD1 pretreatment reduced p-p65 and p-IκBα levels and attenuated LPS-induced IκBα degradation (**Figure 1A**). Time-course analysis showed that inhibition of LPS-induced NF-κB activation by RvD1 was most pronounced at 1 h (**Figures 1B–D**). LPS increased *IL6* and *IL8* mRNA expression, which peaked at 8 h, whereas RvD1 pretreatment significantly reduced the LPS-induced increases in their mRNA levels (**Figures 1E, F**). IL-6 and IL-8 concentrations in culture supernatants increased over time after LPS stimulation; RvD1 pretreatment reduced secretion of both cytokines at each post-stimulation time point, with the greatest inhibition at 48 h (**Figures 1G, H**). Based on these kinetics, subsequent experiments assessed NF-κB-related proteins at 1 h, cytokine mRNA at 8 h, and cytokine secretion at 48 h.

**Figure 1:**
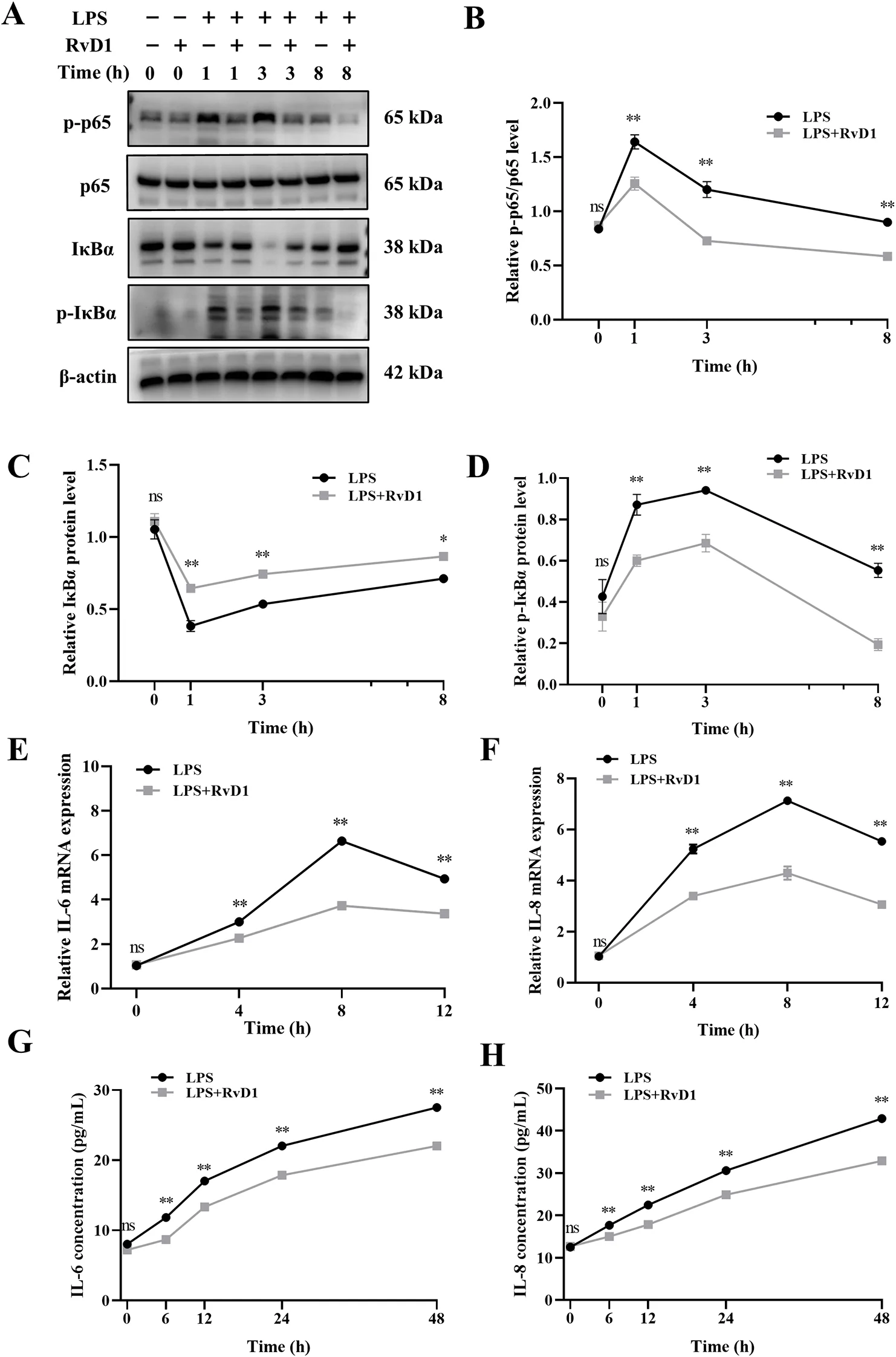
RvD1 attenuates early LPS-induced signaling and late inflammatory output. **(A)** Western blot analysis of p-p65, p65, IκBα, p-IκBα, and β-actin in bovine mammary epithelial cells pretreated with RvD1 for 1 h and then stimulated with LPS for 0, 1, 3, or 8 h. Densitometric signals were normalized to the appropriate loading control or corresponding total protein, as indicated in each panel. **(B–D)** Densitometric quantification of band intensities from three independent experiments. **(E, F)** RT-qPCR analysis of *IL6* and *IL8* mRNA expression after RvD1 pretreatment for 1 h followed by LPS stimulation for 0, 4, 8, or 12 h. **(G, H)** ELISA of IL-6 and IL-8 concentrations in culture supernatants after RvD1 pretreatment for 1 h followed by LPS stimulation for 0, 6, 12, 24, or 48 h. Data are presented as the mean ± SEM. Asterisks indicate comparisons between treatment groups at the same time point. ns, not significant; *P < 0.05; **P < 0.01.

### FPR2 contributes to the RvD1-mediated suppression of LPS-induced NF-κB activation and cytokine production

To assess the role of FPR2 in the anti-inflammatory effect of RvD1, cells were treated with the FPR2 antagonist WRW4. Relative to the LPS + RvD1 group, WRW4 partially reversed the RvD1-induced reductions in p-p65 and p-IκBα and diminished the RvD1-mediated preservation of IκBα. RT-qPCR and ELISA further showed that WRW4 significantly attenuated the inhibition of LPS-induced *IL6* and *IL8* mRNA expression and protein secretion by RvD1 (**Figures 2A–H**). Immunofluorescence suggested an apparent redistribution of FPR2 and ARRB2 and greater spatial overlap between their fluorescence signals; these changes were less apparent in the presence of WRW4 (**Figures 2I, J**).

**Figure 2:**
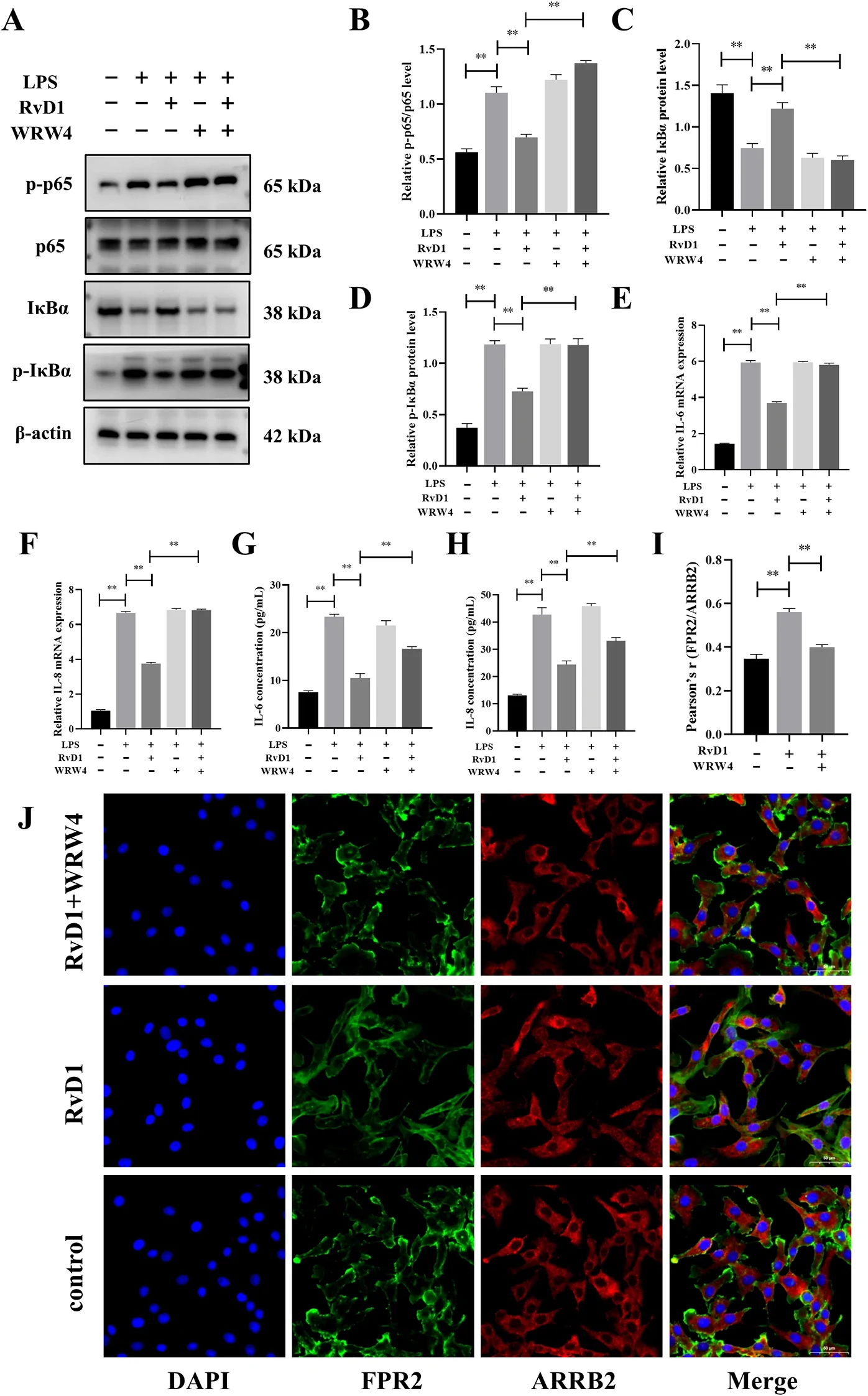
FPR2 contributes to RvD1-mediated suppression of NF-κB activation and cytokine production. **(A)** Western blot analysis of IκBα, p-p65, p65, p-IκBα, and β-actin in cells treated with RvD1 and/or the FPR2 antagonist WRW4 and then stimulated with LPS for 1 h. Densitometric signals were normalized to the appropriate loading control or corresponding total protein, as indicated in each panel. **(B–D)** Densitometric quantification of band intensities from three independent experiments. **(E, F)** RT-qPCR analysis of relative *IL6* and *IL8* mRNA expression in cells treated with RvD1 and/or WRW4 and then stimulated with LPS for 8 h. **(G, H)** ELISA of IL-6 and IL-8 concentrations in culture supernatants from cells treated with RvD1 and/or WRW4 and then stimulated with LPS for 48 h. **(I)** Quantitative analysis of FPR2–ARRB2 colocalization using Pearson’s correlation coefficient from three independent experiments. **(J)** Immunofluorescence analysis of FPR2 and ARRB2 expression and localization after treatment with RvD1 and/or WRW4. Nuclei were stained with DAPI; FPR2 and ARRB2 are shown in green and red, respectively. Scale bar, 50 μm. Data are presented as the mean ± SEM; statistical analyses were performed as described in the Materials and Methods. ns, not significant; *P < 0.05; **P < 0.01.

### ARRB2 mediates the RvD1-induced suppression of NF-κB activation and cytokine production

Immunofluorescence in **Figure 2J** suggested increased spatial overlap between FPR2 and ARRB2 fluorescence signals after RvD1 treatment. To determine whether ARRB2 contributes to the anti-inflammatory activity of RvD1, three ARRB2-targeting siRNAs were tested, and siARRB2-3, which produced the greatest reduction in ARRB2 protein abundance, was selected for subsequent experiments (**Supplementary Figure S2A**). Compared with RvD1 pretreatment, *ARRB2* knockdown restored p-p65 and p-IκBα levels and enhanced IκBα degradation (**Figures 3A–D**). *ARRB2* knockdown also attenuated the inhibitory effects of RvD1 on *IL6* and *IL8* mRNA expression and protein secretion (**Figures 3E–H**). Co-immunoprecipitation showed that RvD1 enhanced FPR2–ARRB2 and ARRB2–IκBα associations, whereas WRW4 weakened both associations (**Figures 3I–L**). LPS increased K48-linked polyubiquitination of IκBα, which was reduced by RvD1 pretreatment; *ARRB2* knockdown attenuated this RvD1-mediated reduction (**Figures 3M, N**).

**Figure 3:**
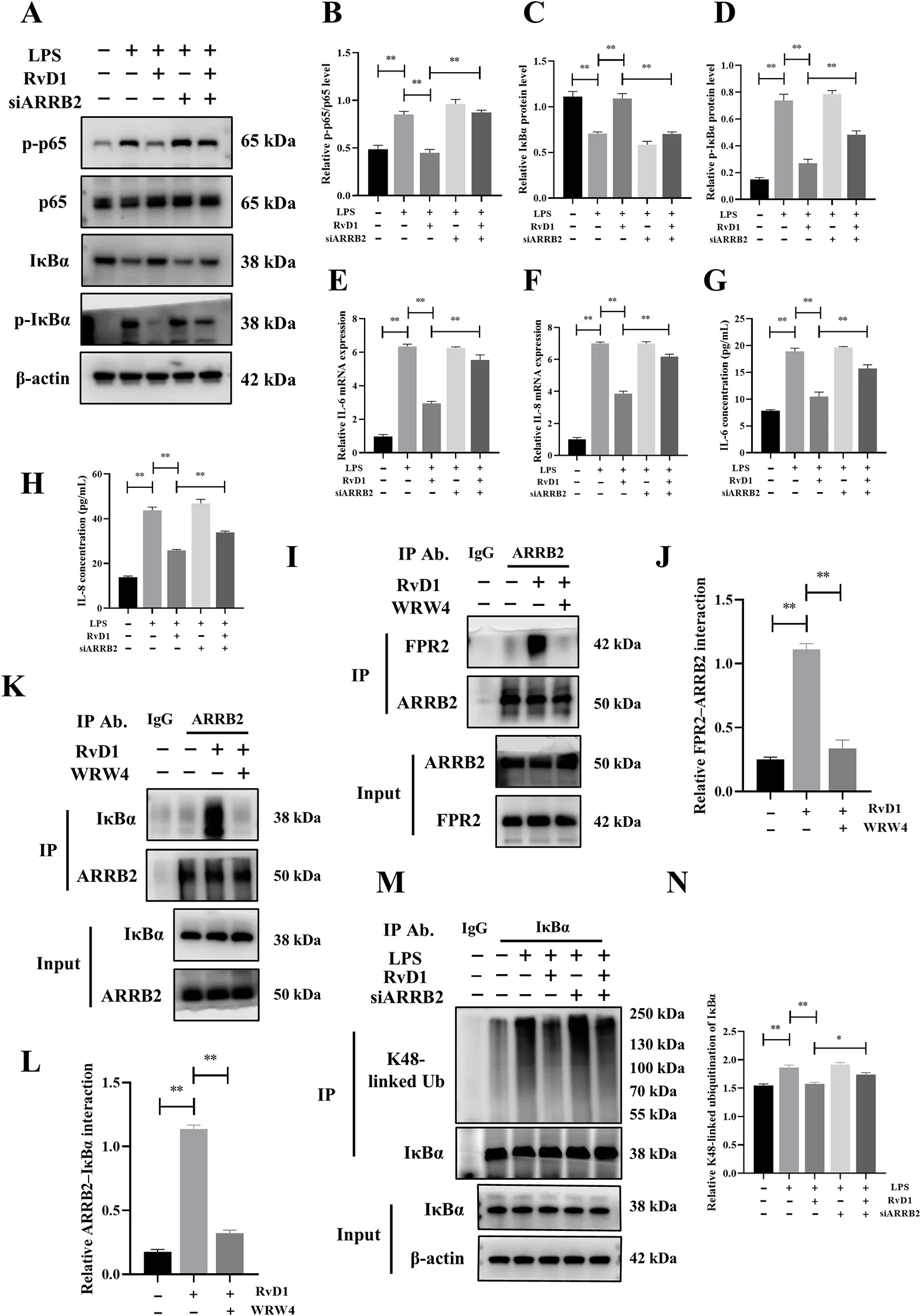
RvD1 signals through FPR2–ARRB2 to suppress early p65 phosphorylation/IκBα degradation and late IL-6/IL-8 production. **(A)** Cells were transfected with siARRB2 for 48 h and/or pretreated with RvD1 before LPS stimulation for 1 h. IκBα, p-p65, p65, p-IκBα, and β-actin were analyzed by Western blotting. Signals were normalized to the appropriate loading control or corresponding total protein, as indicated. **(B–D)** Densitometric quantification of p-p65, IκBα, and p-IκBα from three independent experiments. **(E, F)** RT-qPCR analysis of *IL6* and *IL8* mRNA in cells transfected with siARRB2 for 48 h and/or pretreated with RvD1 before LPS stimulation for 8 h. **(G, H)** ELISA of IL-6 and IL-8 concentrations in culture supernatants after the same transfection and pretreatment followed by LPS stimulation for 48 h. **(I–L)** Cells were treated with RvD1 for 1 h, with WRW4 added 1 h before RvD1 where indicated. Lysates were immunoprecipitated with an anti-ARRB2 antibody and immunoblotted for FPR2 **(I, J)** or IκBα **(K, L)**. IgG served as the negative control. Co-immunoprecipitated proteins were normalized to the corresponding immunoprecipitated bait protein. **(M, N)** Cells were transfected with siARRB2 or siNC for 48 h and treated with MG132 (10 μM), which was added 2 h before RvD1 pretreatment and maintained during subsequent RvD1 and LPS treatments (4 h total). After RvD1 pretreatment for 1 h and LPS stimulation for 1 h, IκBα was immunoprecipitated, and K48-linked polyubiquitination was analyzed by Western blotting and normalized to immunoprecipitated IκBα. In the siARRB2 row, “−” indicates siNC and “+” indicates siARRB2. Data are mean ± SEM; statistical analyses were performed as described in the Materials and Methods. ns, not significant; *P < 0.05; **P < 0.01. IP, immunoprecipitation; IB, immunoblotting; siNC, negative-control siRNA.

### RvD1 upregulates *DLK1–DIO3* cluster miRNAs and is associated with inflammatory negative-feedback regulation

To identify RvD1-associated changes in miRNA expression, small RNA sequencing was performed in control (CK), LPS-stimulated (L), and RvD1-pretreated plus LPS-stimulated (RL) cells. Differentially expressed miRNAs were defined by |log_2_FC| > 1 and FDR < 0.05. Relative to the control group, LPS treatment resulted in 49 differentially expressed miRNAs (16 upregulated and 33 downregulated). Relative to the control group, RvD1 + LPS treatment resulted in 113 differentially expressed miRNAs (47 upregulated and 66 downregulated). Relative to the LPS group, RvD1 + LPS treatment resulted in 103 differentially expressed miRNAs (52 upregulated and 51 downregulated) (**Figures 4A–C**, respectively). Venn analysis revealed no miRNA shared by all three comparisons; most differentially expressed miRNAs were unique to individual comparisons or shared between two comparisons (**Figure 4D**). Hierarchical clustering showed that several miRNAs within the *DLK1–DIO3* imprinted region were not appreciably induced by LPS alone but were coordinately upregulated by RvD1 pretreatment in the presence of LPS, including bta-miR-380-5p, bta-miR-654, bta-miR-381, bta-miR-379, bta-miR-411a, bta-miR-431, bta-miR-370, bta-miR-432, and bta-miR-493 (**Figure 4E**). These miRNAs were highly correlated in their expression patterns (**Figure 4F**). GO analysis showed that their predicted target genes were enriched in regulation of interleukin-8 production, regulation of cytokine production, negative regulation of cytokine production, chemokine-mediated signaling pathway, Toll-like receptor signaling pathway, inflammatory response, cytokine-mediated signaling pathway, regulation of MAPK cascade, pattern recognition receptor signaling pathway, and regulation of chemokine production (**Figure 4G**). KEGG analysis identified enrichment in regulation of actin cytoskeleton, leukocyte transendothelial migration, cytokine–cytokine receptor interaction, bacterial invasion of epithelial cells, efferocytosis, lysosome, TNF signaling pathway, chemokine signaling pathway, and endocytosis (**Figure 4H**). The predicted miRNA–target network linked multiple DLK1–DIO3 miRNAs to inflammation-related genes, including TAB2, IL-33, TRAF6, TLR4, MAPK14, and TNF (**Figure 4I**). bta-miR-380-5p and bta-miR-654 were prominently upregulated in the RL group and were predicted to target TAB2 and IL-33, respectively; these two miRNA–target pairs were therefore selected for validation. Genomic annotation placed MEG3 and the RvD1-responsive miRNAs within the bovine chromosome 21 *DLK1–DIO3* imprinted region (**Figure 4J**). RT-qPCR showed that LPS alone reduced *MEG3* expression but did not increase the selected DLK1–DIO3 miRNAs. RvD1 treatment alone increased the expression of several selected miRNAs, whereas RvD1 pretreatment followed by LPS stimulation produced the most consistent increases in MEG3 and the examined miRNAs (**Figure 4K**).

**Figure 4:**
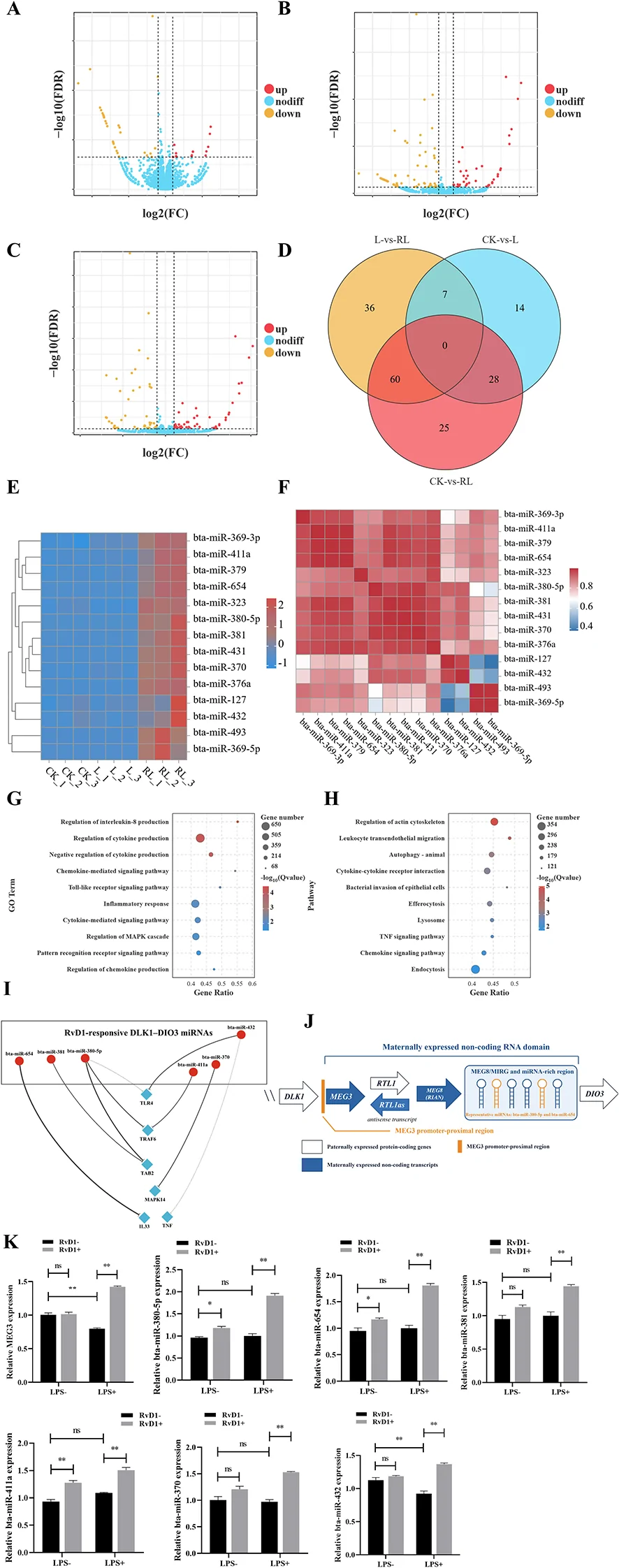
RvD1 induces *DLK1–DIO3* cluster miRNAs and reveals a putative inflammation-related target network. **(A–C)** Small RNA sequencing and volcano-plot analysis of differentially expressed miRNAs in untreated control, LPS-treated, and RvD1 + LPS-treated cells. LPS-treated cells were stimulated for 12 h; RvD1 + LPS-treated cells were pretreated with RvD1 for 1 h before LPS stimulation for 12 h. **(A)** CK-vs-L (LPS relative to untreated control); **(B)** CK-vs-RL (RvD1 + LPS relative to untreated control); **(C)** L-vs-RL (RvD1 + LPS relative to LPS). **(D)** Venn analysis of differentially expressed miRNAs across comparisons. **(E)** Hierarchical-clustering heatmap of candidate RvD1-responsive *DLK1–DIO3* cluster miRNAs. **(F)** Correlation matrix of candidate *DLK1–DIO3* cluster miRNAs across nine sequenced samples; colors indicate correlation coefficients. **(G, H)** GO biological-process and KEGG pathway enrichment analyses of predicted target genes. **(I)** Regulatory network of candidate *DLK1–DIO3* cluster miRNAs and inflammation-related target genes based on TargetScan and miRanda predictions; interactions predicted by at least one algorithm were retained. Red circles, miRNAs; blue diamonds, predicted target genes; edges, predicted miRNA–mRNA interactions. Edge thickness reflects miRanda-predicted binding free energy, with thicker edges indicating more negative values and greater predicted duplex stability. **(J)** Simplified schematic of the bovine DLK1–DIO3 imprinted locus. The *MEG3* promoter-proximal region was used to assess the epigenetic state associated with transcription of the maternally expressed non-coding RNA domain. **(K)** Cells were pretreated with RvD1 or vehicle for 1 h and then stimulated with LPS or vehicle for 12 h. MEG3 and candidate *DLK1–DIO3* cluster miRNAs were analyzed by RT-qPCR. Data are mean ± SEM; statistical analyses were performed as described in the Materials and Methods. ns, not significant; *P < 0.05; **P < 0.01. CK, untreated control; R, RvD1-treated; L, LPS-treated; RL, RvD1-pretreated plus LPS-treated; GO, Gene Ontology; KEGG, Kyoto Encyclopedia of Genes and Genomes.

### bta-miR-380-5p targets TAB2 and contributes to the late-phase suppression of inflammatory cytokines by RvD1

Based on the miRNA–target prediction results, TAB2 was selected for further analysis. RT-qPCR showed that transfection with the bta-miR-380-5p mimic markedly increased bta-miR-380-5p expression compared with mimic NC transfection (**Supplementary Figure S2B**). The bta-miR-380-5p mimic reduced LPS-induced TAB2 protein expression, whereas the bta-miR-380-5p inhibitor partially attenuated the reduction in TAB2 protein expression following RvD1 treatment (**Figures 5A–D**). TAB2-WT and TAB2-MUT dual-luciferase reporters were generated based on the predicted bta-miR-380-5p-binding site in the *TAB2* 3′ UTR (**Supplementary Figure S2G**). The bta-miR-380-5p mimic significantly reduced the luciferase activity of TAB2-WT but had no significant effect on TAB2-MUT (**Figure 5E**).

**Figure 5:**
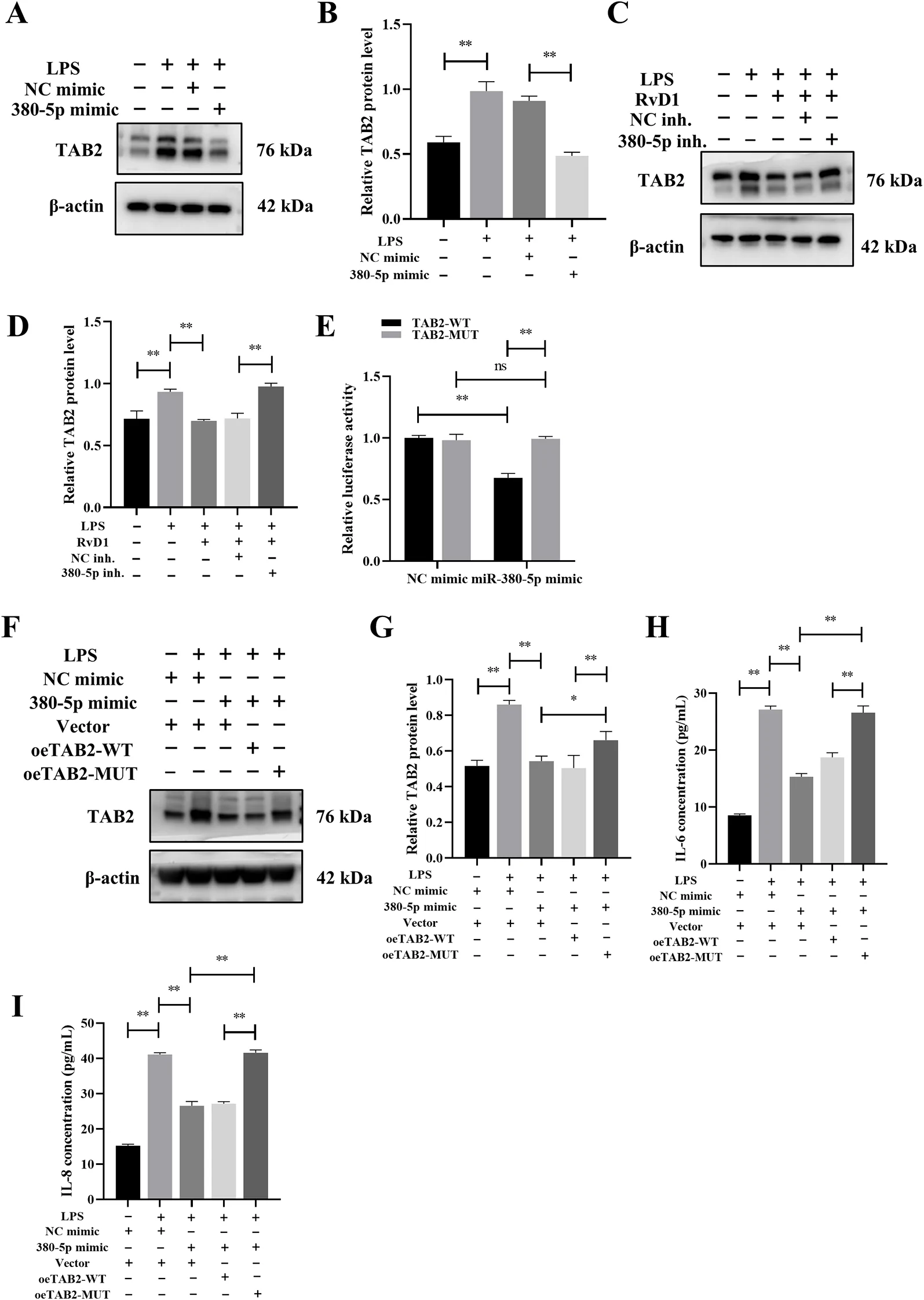
bta-miR-380-5p targets TAB2 and contributes to the RvD1-mediated suppression of inflammatory cytokines. **(A, B)** Cells were transfected with mimic NC or bta-miR-380-5p mimic where indicated for 48 h and stimulated with LPS or vehicle for 36 h before TAB2 protein analysis and densitometric quantification. **(C, D)** Cells were transfected with inhibitor NC or bta-miR-380-5p inhibitor for 48 h, pretreated with RvD1 for 1 h, and stimulated with LPS or vehicle for 36 h before TAB2 protein analysis and densitometric quantification. **(E)** Cells were co-transfected with mimic NC or bta-miR-380-5p mimic and the TAB2-WT or TAB2-MUT dual-luciferase reporter for 48 h before relative luciferase activity measurement. **(F, G)** Cells were co-transfected with the indicated miRNA mimic and empty vector, oeTAB2-WT, or oeTAB2-MUT for 48 h and subsequently stimulated with LPS or vehicle for 36 h before TAB2 protein analysis and densitometric quantification. **(H, I)** Parallel cultures receiving the same transfections were stimulated with LPS or vehicle for 48 h before ELISA analysis of IL-6 and IL-8 secretion. β-actin served as the loading control. NC inh. and 380-5p inh. denote inhibitor NC and bta-miR-380-5p inhibitor, respectively; NC mimic and 380-5p mimic denote mimic NC and bta-miR-380-5p mimic, respectively. TAB2-WT and TAB2-MUT denote the dual-luciferase reporter constructs, whereas oeTAB2-WT and oeTAB2-MUT denote the corresponding TAB2 expression constructs. Data are mean ± SEM from three independent experiments; statistical analyses were performed as described in the Materials and Methods. ns, not significant; *P < 0.05; **P < 0.01.

Rescue experiments were subsequently performed using TAB2 expression constructs containing the wild-type or mutant bta-miR-380-5p-binding site. In vector-transfected cells, the bta-miR-380-5p mimic reduced TAB2 protein abundance and LPS-induced IL-6 and IL-8 secretion. Compared with the vector and oeTAB2-WT groups, oeTAB2-MUT increased TAB2 protein abundance and IL-6 and IL-8 secretion in the presence of the bta-miR-380-5p mimic and LPS (**Figures 5F–I**).

### bta-miR-654 targets IL-33 and contributes to the late-phase suppression of inflammatory cytokines by RvD1

IL-33 was selected for further analysis based on the predicted miRNA–target network. RT-qPCR confirmed that mimic transfection markedly increased bta-miR-654 expression (**Supplementary Figure S2C**). The bta-miR-654 mimic reduced LPS-induced IL-33 protein abundance, whereas its inhibitor attenuated the RvD1-mediated reduction in IL-33 under LPS stimulation (**Figures 6A–D**). Dual-luciferase reporters containing the predicted bta-miR-654-binding site or its mutant sequence in the *IL-33* 3′ UTR were then examined (**Supplementary Figure S2G**). The mimic reduced the luciferase activity of IL-33-WT but did not affect IL-33-MUT (**Figure 6E**).

**Figure 6:**
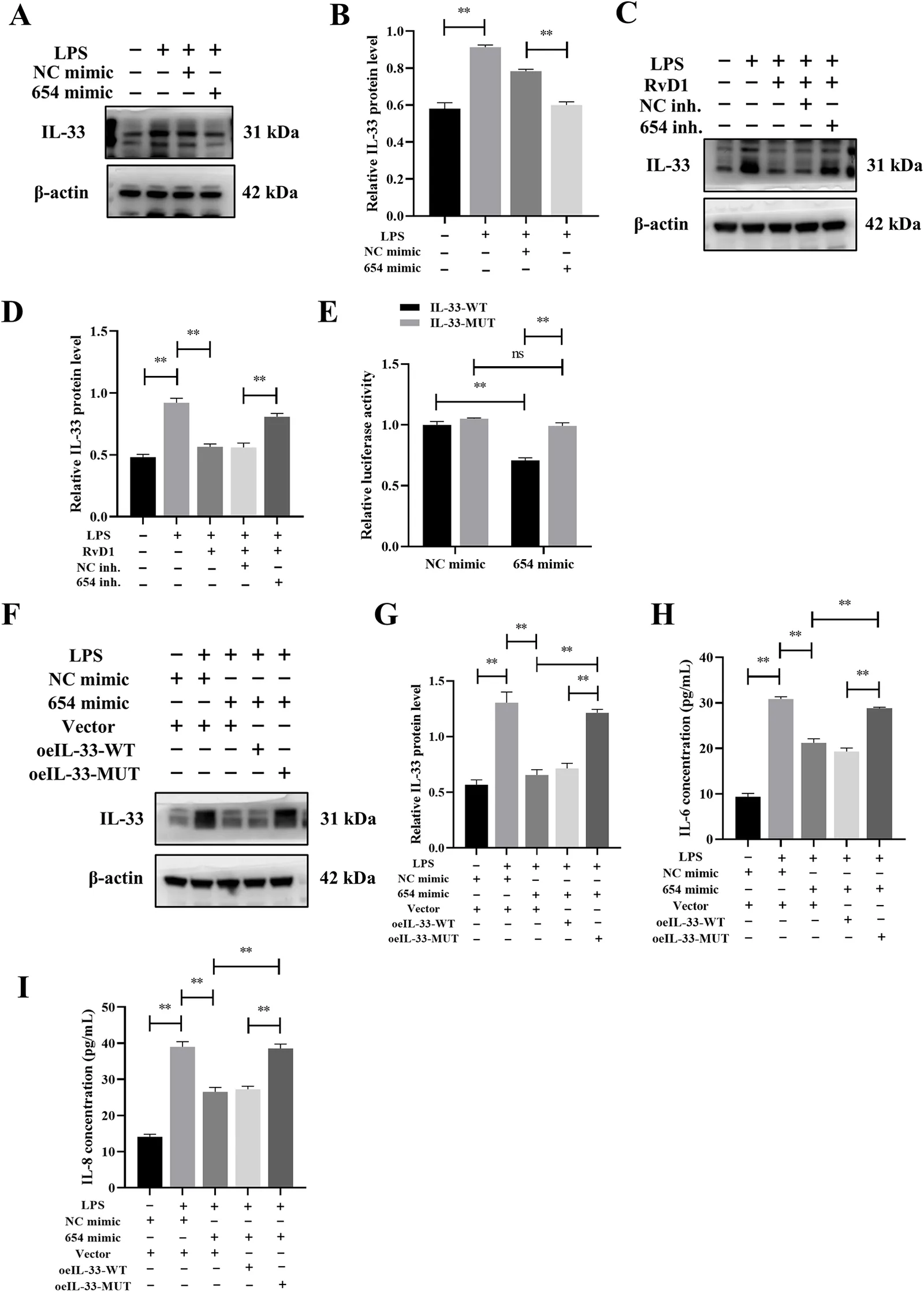
bta-miR-654 targets IL-33 and contributes to the RvD1-mediated suppression of inflammatory cytokines. **(A, B)** Cells were transfected with mimic NC or bta-miR-654 mimic where indicated for 48 h and stimulated with LPS or vehicle for 36 h before IL-33 protein analysis and densitometric quantification. **(C, D)** Cells were transfected with inhibitor NC or bta-miR-654 inhibitor for 48 h, pretreated with RvD1 for 1 h, and stimulated with LPS or vehicle for 36 h before IL-33 protein analysis and densitometric quantification. **(E)** Cells were co-transfected with mimic NC or bta-miR-654 mimic and the IL-33-WT or IL-33-MUT dual-luciferase reporter for 48 h before relative luciferase activity measurement. **(F, G)** Cells were co-transfected with the indicated miRNA mimic and empty vector, oeIL-33-WT, or oeIL-33-MUT for 48 h and subsequently stimulated with LPS or vehicle for 36 h before IL-33 protein analysis and densitometric quantification. **(H, I)** Parallel cultures receiving the same transfections were stimulated with LPS or vehicle for 48 h before ELISA analysis of IL-6 and IL-8 secretion. β-actin served as the loading control. NC inh. and 654 inh. denote inhibitor NC and bta-miR-654 inhibitor, respectively; NC mimic and 654 mimic denote mimic NC and bta-miR-654 mimic, respectively. IL-33-WT and IL-33-MUT denote the dual-luciferase reporter constructs, whereas oeIL-33-WT and oeIL-33-MUT denote the corresponding IL-33 expression constructs. Data are mean ± SEM from three independent experiments; statistical analyses were performed as described in the Materials and Methods. ns, not significant; *P < 0.05; **P < 0.01.

For rescue experiments, cells were co-transfected with the bta-miR-654 mimic and the corresponding IL-33 expression constructs. In vector-transfected cells, the mimic reduced IL-33 protein abundance and IL-6/IL-8 secretion following LPS stimulation. Compared with the vector and oeIL-33-WT groups, oeIL-33-MUT increased IL-33 protein abundance and IL-6/IL-8 secretion under the same conditions (**Figures 6F–I**).

### ARRB2 contributes to RvD1-mediated chromatin regulation at the *MEG3* promoter-proximal region

The preceding results showed that *ARRB2* knockdown weakened the inhibitory effects of RvD1 on IκBα degradation and NF-κB activation. We further examined the effect of *ARRB2* knockdown on the RvD1-mediated inhibition of p65 nuclear translocation and analyzed the role of p65 in *MEG3* transcriptional regulation. Nuclear–cytoplasmic fractionation showed that LPS stimulation promoted the accumulation of p65 in the nucleus, whereas RvD1 pretreatment reduced nuclear p65 levels; after *ARRB2* knockdown, the inhibitory effect of RvD1 on nuclear p65 accumulation was weakened (**Figures 7A, B**). Meanwhile, LPS stimulation reduced *MEG3* expression, whereas RvD1 pretreatment significantly upregulated MEG3 and the *DLK1–DIO3* cluster miRNAs bta-miR-380-5p and bta-miR-654. After p65 knockdown (**Supplementary Figure S2D**), the LPS-induced inhibition of *MEG3* expression was alleviated, although *MEG3* expression remained lower than that in the RvD1-pretreated group; however, the expression of bta-miR-380-5p and bta-miR-654 did not increase markedly (**Figures 7C–E**).

**Figure 7:**
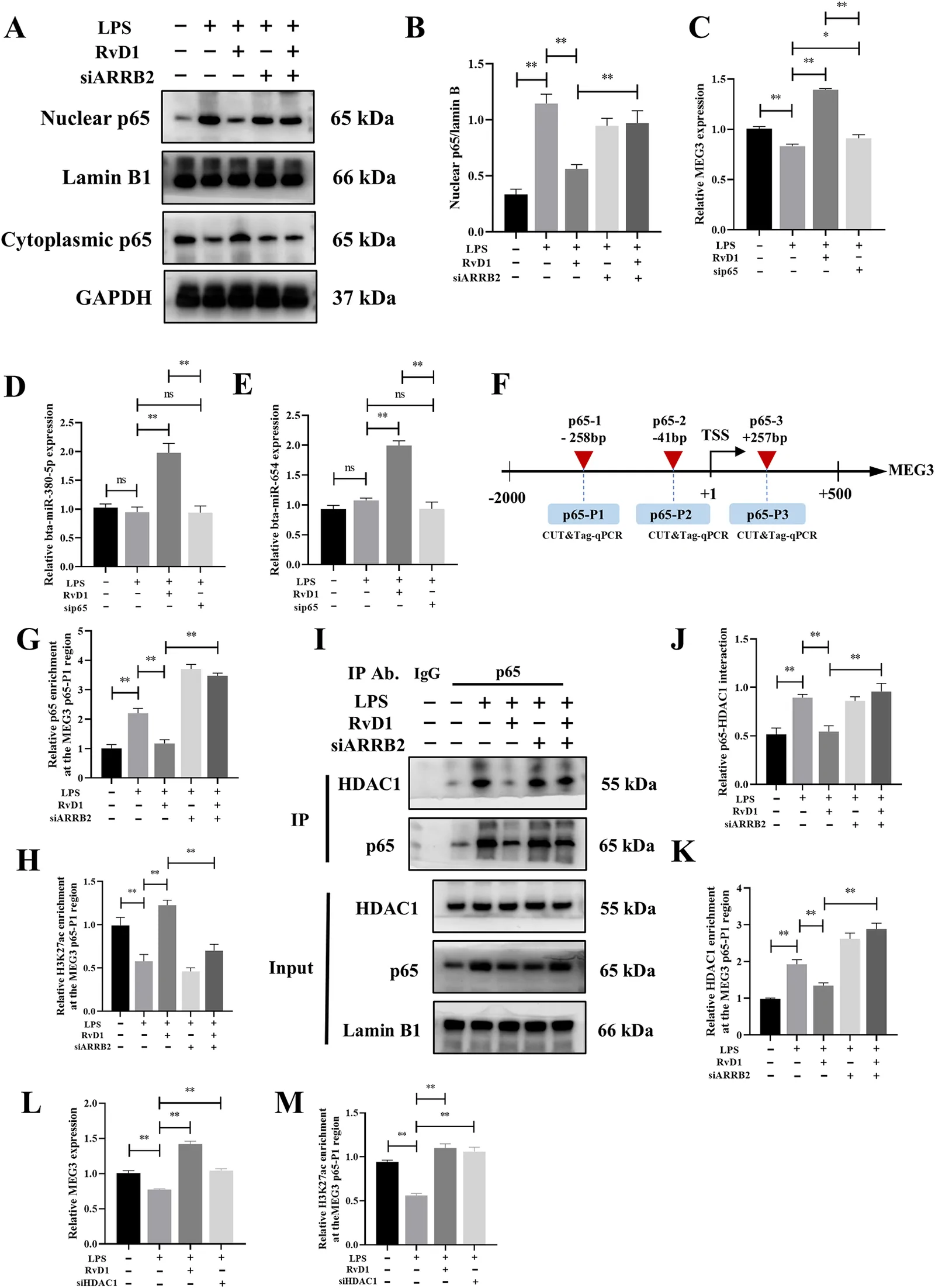
RvD1 reduces p65 and HDAC1 occupancy at the MEG3 region and restores H3K27ac through ARRB2. **(A, B)** Cells were transfected with siARRB2 or siNC for 48 h, pretreated with RvD1 for 1 h, and stimulated with LPS for 1 h. Nuclear and cytoplasmic p65 levels were analyzed using Lamin B1 and GAPDH as the respective loading controls; nuclear p65 was quantified from three independent experiments. **(C–E)** RT-qPCR analysis of MEG3, bta-miR-380-5p, and bta-miR-654 in cells transfected with sip65 or siNC for 48 h and treated with RvD1 and/or LPS; LPS stimulation lasted 12 h. **(F)** RELA-binding sites from −2000 to +500 bp relative to the *MEG3* transcription start site were predicted using JASPAR. Red inverted triangles indicate predicted sites; blue boxes indicate p65 CUT&Tag-qPCR amplicons. **(G, H, K)** CUT&Tag-qPCR analysis at p65-P1 in siARRB2- or siNC-transfected cells pretreated with RvD1 for 1 h. p65 **(G)** and HDAC1 **(K)** enrichment were assessed after 1 h LPS stimulation, and H3K27ac enrichment **(H)** after 8 h. IgG was the negative control. **(I, J)** Cells were transfected with siARRB2 or siNC for 48 h, pretreated with RvD1 for 1 h, and stimulated with LPS for 1 h. Nuclear extracts were immunoprecipitated with anti-p65 and immunoblotted for HDAC1. IgG served as the negative control, and Lamin B1 was used as the nuclear loading control. The p65–HDAC1 interaction was quantified from three independent experiments, with co-immunoprecipitated HDAC1 normalized to immunoprecipitated p65. **(L, M)** In cells transfected with siHDAC1 or siNC for 48 h, *MEG3* expression was analyzed after 12 h LPS stimulation **(L)**, and H3K27ac enrichment at p65-P1 after 8 h **(M)**. IgG was the CUT&Tag negative control. In siRNA rows, “−” indicates siNC and “+” indicates the corresponding target siRNA. Data are mean ± SEM; statistics were performed as described in Materials and Methods. ns, not significant; *P < 0.05; **P < 0.01.

JASPAR prediction identified multiple potential p65-binding sites in the *MEG3* promoter-proximal region (**Supplementary Table S1A**). Based on the positions of the predicted sites, p65-P1, p65-P2, and p65-P3 regions were designed for CUT&Tag-qPCR analysis (**Figure 7F**). Among the three regions examined, significant treatment-associated changes in p65 enrichment were detected at p65-P1. LPS stimulation increased p65 enrichment at p65-P1, whereas RvD1 pretreatment reduced its enrichment; *ARRB2* knockdown weakened the inhibitory effect of RvD1 on p65 enrichment (**Figure 7G**). At the same region, RvD1 increased H3K27ac enrichment under LPS stimulation, whereas *ARRB2* knockdown weakened the restorative effect of RvD1 on H3K27ac (**Figure 7H**). STRING protein-interaction prediction indicated potential interactions between p65 and HDAC1, HDAC2, and HDAC3 (**Supplementary Figure S2H**). We further examined the nuclear p65–HDAC1 interaction and HDAC1 enrichment at the MEG3 region under LPS and RvD1 treatment. LPS stimulation enhanced the nuclear p65–HDAC1 interaction, whereas RvD1 reduced this interaction; *ARRB2* knockdown weakened the inhibitory effect of RvD1 (**Figures 7I, J**). CUT&Tag-qPCR showed that RvD1 reduced LPS-induced HDAC1 enrichment at p65-P1, whereas *ARRB2* knockdown weakened this effect (**Figure 7K**). In addition, siHDAC1 effectively reduced HDAC1 protein expression (**Supplementary Figure S2E**); under this condition, siHDAC1 alleviated the LPS-induced decrease in *MEG3* expression and increased H3K27ac enrichment at p65-P1 (**Figures 7L, M**).

### RvD1 activates CREB and promotes the expression of MEG3 and *DLK1–DIO3* cluster miRNAs

RvD1 treatment alone increased CREB phosphorylation over time (**Figures 8A, B**); in the presence of LPS, RvD1 treatment further increased p-CREB levels, whereas WRW4 weakened this effect (**Figures 8C, D**). Potential CREB1-binding sites in the bovine MEG3 regulatory region were predicted using JASPAR (**Supplementary Table S1B**), and two CUT&Tag-qPCR regions, CREB1-P1 and CREB1-P2, were designed accordingly (**Figure 8E**). Of these regions, significant treatment-associated changes in CREB1 enrichment were detected at CREB1-P1. RvD1 treatment significantly increased CREB1 enrichment at CREB1-P1, whereas *CREB1* knockdown markedly reduced RvD1-induced CREB1 enrichment (**Figure 8F**; knockdown efficiency is shown in **Figure S2F**). RvD1 also upregulated MEG3, bta-miR-380-5p, and bta-miR-654 expression; *CREB1* knockdown weakened the RvD1-mediated upregulation of MEG3 and these miRNAs (**Figures 8G–I**). Further analysis of downstream inflammation-related molecules showed that RvD1 reduced LPS-induced TAB2 and IL-33 protein expression and inhibited *IL6* and *IL8* mRNA expression and protein secretion, whereas *CREB1* knockdown weakened all of these regulatory effects (**Figures 8J–P**).

**Figure 8:**
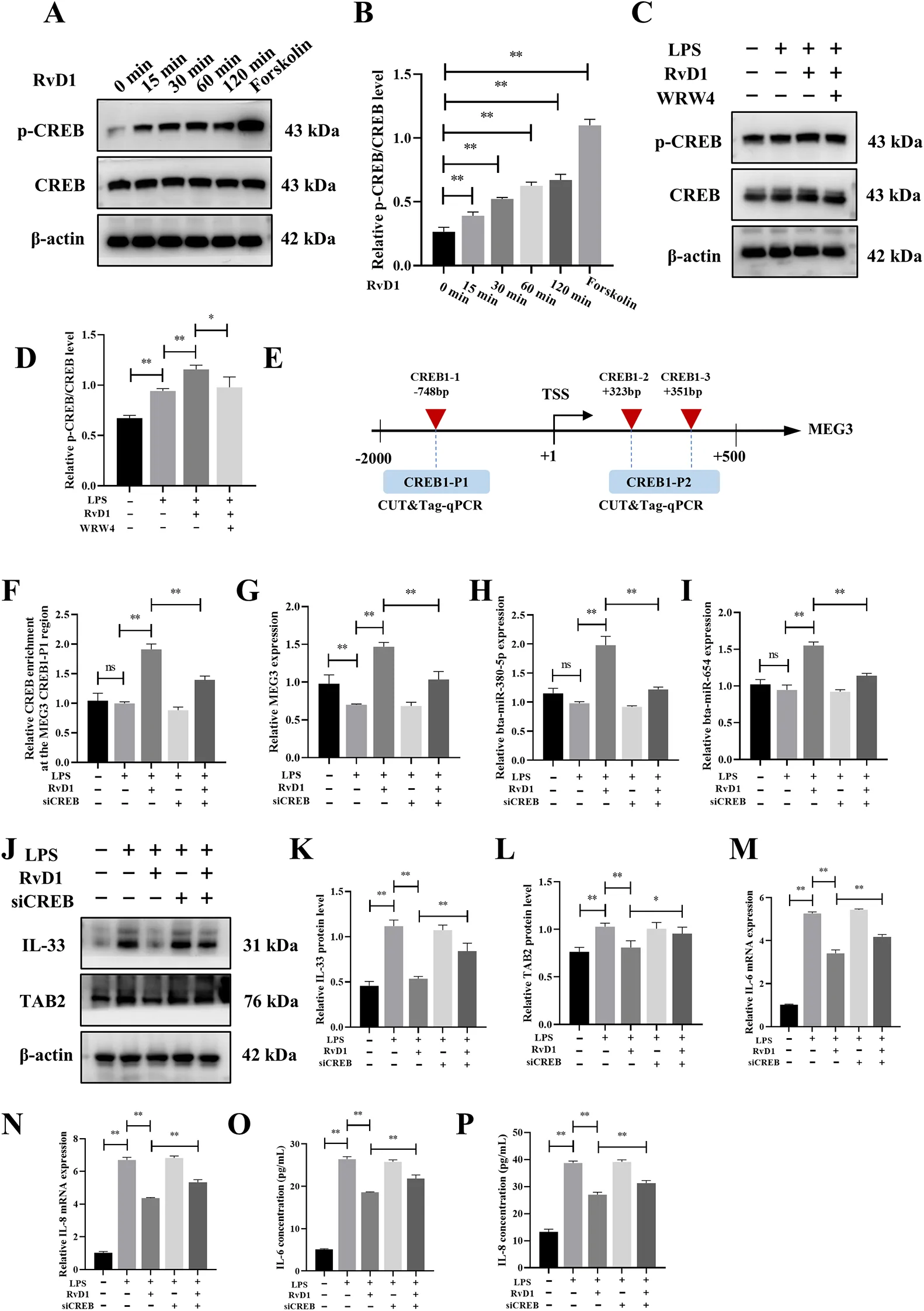
RvD1 activates CREB and promotes the expression of MEG3 and *DLK1–DIO3* cluster miRNAs. **(A, B)** p-CREB and total CREB were immunoblotted after RvD1 treatment for 0, 15, 30, 60, or 120 min; Forskolin was the positive control. Signals were normalized to the appropriate loading control or total protein. p-CREB was quantified from three experiments and normalized to total CREB. **(C, D)** Cells were pretreated with WRW4 and RvD1 for 1 h each before 1 h LPS stimulation. p-CREB and total CREB were immunoblotted; β-actin was the loading control. p-CREB was quantified from three experiments and normalized to total CREB. **(E)** CREB1-binding sites from −2000 to +500 bp relative to the *MEG3* transcription start site were predicted using JASPAR. Red inverted triangles indicate predicted sites; blue boxes indicate CUT&Tag-qPCR amplicons. **(F)** CUT&Tag-qPCR analysis of CREB1 enrichment at CREB1-P1 in cells transfected with siCREB or siNC for 48 h, pretreated with RvD1 for 1 h, and stimulated with LPS for 1 h. IgG was the negative control. **(G–I)** RT-qPCR analysis of MEG3, bta-miR-380-5p, and bta-miR-654 after the same transfection and pretreatment, followed by 12 h LPS stimulation. **(J–L)** IL-33 and TAB2 were immunoblotted and quantified after 36 h LPS stimulation under the same conditions. β-actin was the loading control; signals from three experiments were normalized to β-actin. **(M, N)** RT-qPCR analysis of *IL6* and *IL8* mRNA after 8 h LPS stimulation under the same conditions. **(O, P)** ELISA of IL-6 and IL-8 in culture supernatants after 48 h LPS stimulation under the same conditions. Data are mean ± SEM; statistics were performed as described in Materials and Methods. ns, not significant; *P < 0.05; **P < 0.01. In the siCREB row, “−” indicates siNC and “+” indicates siCREB.

### RvD1 induces a DLK1–DIO3 miRNA-associated response in primary bovine mammary epithelial cells

To determine whether the RvD1-associated response observed in MAC-T cells was also present in primary bovine mammary epithelial cells, primary cells were treated with RvD1 and/or LPS. RvD1 pretreatment reduced LPS-induced p-p65 and p-IκBα levels and attenuated the decrease in IκBα protein abundance (**Figures 9A–D**). LPS reduced *MEG3* expression without significantly altering bta-miR-380-5p or bta-miR-654, whereas RvD1 pretreatment increased all three under LPS stimulation (**Figures 9E–G**). RvD1 pretreatment also reduced the LPS-induced increases in TAB2 and IL-33 protein abundance and IL-6/IL-8 secretion (**Figures 9H–L**).

**Figure 9:**
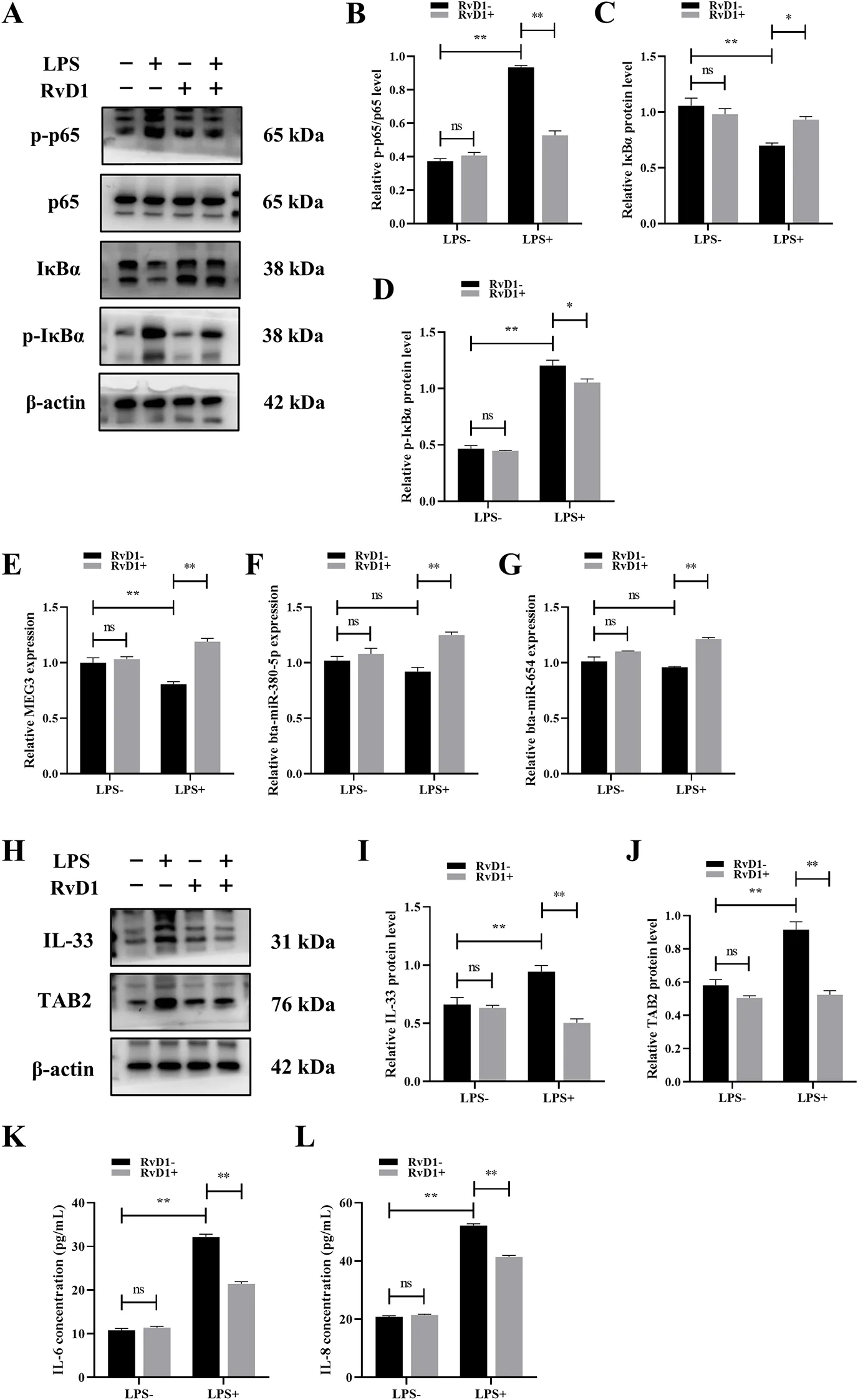
RvD1 suppresses early NF-κB activation and induces a later DLK1–DIO3 miRNA-associated anti-inflammatory response in primary bovine mammary epithelial cells. Primary bovine mammary epithelial cells were pretreated with 100 nM RvD1 or vehicle for 1 h and then stimulated with 1 μg/mL LPS or vehicle. **(A)** Western blot analysis of p-p65, p65, IκBα, and p-IκBα after 1 h of LPS stimulation; β-actin served as the loading control. **(B–D)** Densitometric quantification of the p-p65/p65 ratio and IκBα and p-IκBα protein abundance. **(E–G)** RT-qPCR analysis of MEG3, bta-miR-380-5p, and bta-miR-654 after 12 h of LPS stimulation. **(H)** Western blot analysis of IL-33 and TAB2 after 36 h of LPS stimulation; β-actin served as the loading control. **(I, J)** Densitometric quantification of IL-33 and TAB2 protein abundance. **(K, L)** IL-6 and IL-8 concentrations in culture supernatants measured by ELISA after 48 h of LPS stimulation. All quantitative data are presented as the mean ± SEM from three independent donors (n = 3). Statistical analyses were performed as described in the Materials and Methods. ns, not significant; *P < 0.05; **P < 0.01.

## Discussion

Here, an LPS-induced bovine mammary epithelial inflammation model was used to evaluate the effects of RvD1 on early NF-κB activation, intermediate cytokine transcription, and later cytokine secretion. RvD1 pretreatment reduced LPS-induced p-p65 and p-IκBα levels, attenuated IκBα degradation, and decreased IL-6 and IL-8 expression and secretion. Given the pretreatment design, the present findings reflect the ability of RvD1 to attenuate subsequent LPS-induced inflammatory activation. Formyl peptide receptor 2 (ALX/FPR2) is a G protein-coupled receptor with important functions in host defense and inflammation. Depending on the bound agonist, FPR2 can mediate either pro-inflammatory or anti-inflammatory responses (30). Pharmacological inhibition of FPR2 with WRW4 weakened the ability of RvD1 to suppress LPS-induced inflammation in bovine mammary epithelial cells, indicating that this effect is at least partly FPR2 dependent. Consistent with this observation, Nam et al. reported that aspirin-triggered RvD1 activation of human FPR2 alleviated LPS-induced acute sialadenitis, reduced inflammatory-cell proliferation, and restored salivary secretion (31). RvD1–FPR2 signaling has also been reported to attenuate inflammation through a Rac1/NOX2-related pathway in neonatal hypoxic–ischemic injury (32). ARRB2 signaling downstream of FPR2 is highly dependent on the ligand and cellular context, and different FPR2 agonists vary in their ability to recruit ARRB2, alter receptor trafficking, and elicit cellular responses (33, 34). RvD1 activates the pro-resolving receptors ALX/FPR2 and GPR32 in ARRB2 reporter systems (35), supporting a link between RvD1 and ARRB2-associated G protein-coupled receptor signaling. In the present study, RvD1 treatment was associated with an apparent redistribution of FPR2 and increased spatial overlap between FPR2 and ARRB2 fluorescence signals. Gao et al. showed that β-arrestin 2 directly interacts with IκBα and prevents its phosphorylation and degradation, thereby regulating NF-κB activation and target-gene expression (36). Co-immunoprecipitation further showed that RvD1 enhanced FPR2–ARRB2 and ARRB2–IκBα associations; *ARRB2* knockdown attenuated the ability of RvD1 to reduce IκBα K48-linked polyubiquitination, NF-κB activation, and IL-6/IL-8 expression and secretion.

miRNA regulation is an important component of RvD1-mediated pro-resolving responses (37, 38). RT-qPCR showed that LPS alone did not appreciably induce *DLK1–DIO3* cluster miRNAs, whereas RvD1 alone modestly increased several miRNAs, and these increases were more pronounced in the presence of LPS. Notably, LPS reduced *MEG3* expression without clearly altering mature bta-miR-380-5p or bta-miR-654 at the time examined, indicating that *MEG3* expression and accumulation of these mature miRNAs were not strictly synchronous. In the murine Dlk1–Dio3 region, the *Meg3* promoter drives a long polycistronic transcript containing Meg3, Rian, and Mirg, but internal cis-regulatory elements can selectively regulate downstream miRNA subclusters (39). Moreover, processing of primary miRNAs into mature miRNAs is itself subject to post-transcriptional control, and mature-miRNA abundance therefore need not parallel that of the upstream long transcript (40). By contrast, RvD1 increased MEG3 and the selected miRNAs together, consistent with coordinated activation of the maternal non-coding RNA domain and its associated miRNAs, with the inflammatory context potentially amplifying this response. miRNA-mediated negative feedback through inflammatory signaling nodes is well established. miR-146a/b targets IRAK1 and TRAF6 to restrain Toll-like receptor and cytokine-receptor signaling (38), whereas miR-21 regulates TLR4/NF-κB activity and IL-10 production by targeting PDCD4 (41). In the present study, bta-miR-380-5p reduced TAB2 protein abundance and bta-miR-654 reduced IL-33 protein abundance, whereas expression of the corresponding miRNA-binding-site-mutant constructs restored target-protein abundance and reversed the mimic-induced reductions in IL-6 and IL-8 secretion. Takaesu et al. placed TAB2 between TRAF6 and TAK1 in IL-1 signaling (42), whereas Moussion et al. described IL-33 as a nuclear alarmin associated with epithelial and barrier-related inflammatory responses (43). Together with the early ARRB2–IκBα data, these findings support a temporally layered model in which RvD1 first restrains NF-κB activation and then limits IL-6/IL-8 secretion through the bta-miR-380-5p/TAB2 and bta-miR-654/IL-33 axes.

RvD1 inhibits IκBα degradation and NF-κB p65 nuclear translocation in LPS-induced acute lung injury (44). In LPS-stimulated RAW264.7 macrophages, RvD1 also reduces nuclear accumulation of p65/p50 heterodimers and modulates NF-κB composition in association with pro-resolving functions (45). Our results similarly showed that RvD1 reduced LPS-induced nuclear p65 accumulation and that *ARRB2* knockdown weakened this effect. As a central transcriptional regulator of NF-κB signaling, chromatin-bound p65 can produce different transcriptional outputs depending on its coregulators and the local chromatin environment (46, 47). Direct transcriptional functions of p65 have been demonstrated through its transactivation domains, interaction with the CBP/p300 coactivators, and genome-wide RelA-binding profiles (46). In the present study, LPS increased p65 occupancy at p65-P1, accompanied by reduced *MEG3* expression and lower H3K27ac at this region. RvD1 reduced p65 occupancy, and this effect was weakened by *ARRB2* knockdown. Interactions between p65 and HDAC-containing complexes have been implicated in negative regulation of NF-κB signaling during LPS stimulation (48). For example, ATF3 forms a complex with p65 and recruits HDAC1 in LPS-stimulated RAW264.7 cells (49), whereas c-Ski enhances its interactions with p65 and HDAC1 under LPS stimulation, reduces p65 acetylation, and suppresses TNF-α and IL-1β expression (50). We likewise found that LPS increased both the p65–HDAC1 interaction and HDAC1 occupancy at p65-P1. RvD1 attenuated these changes, whereas *ARRB2* knockdown diminished its effects. Thus, ARRB2 may contribute both to early regulation of the IκBα/NF-κB pathway by RvD1 and to the subsequent control of p65/HDAC1 occupancy at the *MEG3* promoter.

H3K27ac enrichment is commonly used to distinguish active from poised regulatory elements and to characterize active promoters and enhancers (51). In LPS-stimulated nasopharyngeal epithelial cells, H3K27ac ChIP-seq revealed stimulus-induced remodeling of H3K27ac that was associated with RELA binding and gene-expression changes (52). In our study, *HDAC1* knockdown restored H3K27ac at p65-P1 and alleviated the reduction in *MEG3* expression. Analogously, during decidualization of human endometrial stromal cells, C/EBPβ increases H3K27ac at the IGFBP1 and PRL promoters through a p300-associated mechanism, accompanied by increased expression of both genes (53). Targeted increases in H3K27ac using CRISPR/dCas9-p300 epigenome editing can also activate IL1RN and β-globin locus transcripts, including HBE, HBG, and HBD (54).

p65 knockdown partially alleviated the LPS-induced reduction in MEG3 but did not appreciably induce bta-miR-380-5p or bta-miR-654. Thus, p65-associated repression contributes to MEG3 downregulation, but release of this repression alone does not explain the induction of *DLK1–DIO3* cluster miRNAs by RvD1. Aspirin-triggered RvD1 can increase intracellular cAMP and activate PKA signaling through ALX/GPR32 (55), and RvD1 promotes alveolar fluid clearance through an ALX/cAMP-related pathway in alveolar epithelial cells (56), supporting a possible connection between RvD1 and cAMP/PKA-responsive transcription factors. We found that RvD1 increased CREB phosphorylation. Gu et al. similarly observed increased CREB Ser133 phosphorylation in RvD1-treated, TLR4-activated human monocytes and showed that CREB participates in the anti-inflammatory response to RvD1 (57). In our model, WRW4 attenuated RvD1-induced CREB phosphorylation in the presence of LPS. CREB Ser133 phosphorylation promotes recruitment of coactivators such as CBP/p300 and initiates CRE-dependent transcription (58). At the MEG3 regulatory region, RvD1 increased CREB1 occupancy at CREB1-P1 together with *MEG3* expression. *CREB1* knockdown attenuated the RvD1-induced increases in MEG3, bta-miR-380-5p, and bta-miR-654 and partially restored TAB2 and IL-33 protein expression and IL-6/IL-8 output. These findings support a role for CREB1 in the RvD1-associated induction of MEG3, bta-miR-380-5p, and bta-miR-654 and suggest that CREB activation is not merely a downstream marker of altered inflammation.

In bovine mastitis, excessive activation of inflammatory signaling in mammary epithelial cells can contribute to the amplification and persistence of local inflammation. In the present study, RvD1 not only suppressed early NF-κB activation and reduced IL-6 and IL-8 expression and secretion, but also reduced TAB2 and IL-33 through bta-miR-380-5p and bta-miR-654, respectively, thereby further limiting downstream inflammatory output. These effects of RvD1 in mammary epithelial cells may help limit the excessive amplification and persistence of local inflammation during bovine mastitis. Selected RvD1-associated responses were also observed in primary bovine mammary epithelial cells at the predefined time points. However, because an independent kinetic analysis was not performed in primary cells, these experiments do not establish equivalent response kinetics or peak timing between MAC-T and primary cells. Further *in vivo* studies are required to establish the physiological relevance of these findings in bovine mastitis.

## Conclusions

Collectively, RvD1–FPR2 signaling promoted the ARRB2–IκBα interaction, reduced K48-linked polyubiquitination of IκBα, and limited early NF-κB activation. ARRB2 also contributed to the effects of RvD1 on p65/HDAC1 occupancy and H3K27ac at the *MEG3* promoter-proximal region. In parallel, FPR2-sensitive CREB phosphorylation, together with increased CREB1 occupancy at this region, contributed to the induction of MEG3 and *DLK1–DIO3* cluster miRNAs by RvD1. bta-miR-380-5p and bta-miR-654, in turn, targeted TAB2 and IL-33, respectively, and contributed to the suppression of IL-6 and IL-8 secretion. Finally, the anti-inflammatory response to RvD1 observed in primary bovine mammary epithelial cells provides additional support for the biological relevance of the responses characterized in MAC-T cells. These findings delineate a mechanism through which receptor-proximal signaling, chromatin regulation, and miRNA-mediated post-transcriptional control converge to limit inflammatory output in bovine mammary epithelial cells.

## Data Availability

The raw small RNA sequencing data generated in this study have been deposited in the European Nucleotide Archive (ENA) under accession number PRJEB124412.

## References

[1] Motaung TE Petrovski KR Petzer IMRE Thekisoe O Tsilo TJ . Importance of bovine mastitis in Africa. Anim Health Res Rev. (2017) 18:58–69. doi: 10.1017/S1466252317000032 28606203

[2] Touza-Otero L Landin M Diaz-Rodriguez P . Fighting antibiotic resistance in the local management of bovine mastitis. BioMed Pharmacother. (2024) 170:115967. doi: 10.1016/j.biopha.2023.115967 38043445

[3] Rainard P Gilbert FB Germon P . Immune defenses of the mammary gland epithelium of dairy ruminants. Front Immunol. (2022) 13:1031785. doi: 10.3389/fimmu.2022.1031785 36341445 PMC9634088

[4] Zhao X Lacasse P . Mammary tissue damage during bovine mastitis: causes and control. J Anim Sci. (2008) 86:57–65. doi: 10.2527/jas.2007-0302 17785603

[5] Morales-Ubaldo AL Rivero-Perez N Valladares-Carranza B Velázquez-Ordoñez V Delgadillo-Ruiz L Zaragoza-Bastida A . Bovine mastitis, a worldwide impact disease: Prevalence, antimicrobial resistance, and viable alternative approaches. Vet Anim Sci. (2023) 21:100306. doi: 10.1016/j.vas.2023.100306 37547227 PMC10400929

[6] Serhan CN Savill J . Resolution of inflammation: the beginning programs the end. Nat Immunol. (2005) 6:1191–97. doi: 10.1038/ni1276 16369558

[7] Basil MC Levy BD . Specialized pro-resolving mediators: endogenous regulators of infection and inflammation. Nat Rev Immunol. (2016) 16:51–67. doi: 10.1038/nri.2015.4 26688348 PMC5242505

[8] Serhan CN . Pro-resolving lipid mediators are leads for resolution physiology. Nature. (2014) 510:92–101. doi: 10.1038/nature13479 24899309 PMC4263681

[9] Arnardottir H Thul S Pawelzik SC Karadimou G Artiach G Gallina AL . The resolvin D1 receptor GPR32 transduces inflammation resolution and atheroprotection. J Clin Invest. (2021) 131:e142883. doi: 10.1172/JCI142883 34699386 PMC8670838

[10] Thompson D McArthur S Hislop JN Flower RJ Perretti M . Identification of a novel recycling sequence in the C-tail of FPR2/ALX receptor: association with cell protection from apoptosis. J Biol Chem. (2014) 289:36166–78. doi: 10.1074/jbc.M114.612630 25326384 PMC4276880

[11] Jack CE Cope EM Lemel L Canals M Drube J Hoffmann C . GRK5 regulates endocytosis of FPR2 independent of β-arrestins. J Biol Chem. (2025) 301:108112. doi: 10.1016/j.jbc.2024.108112 39706266 PMC11773488

[12] Kizaki T Izawa T Sakurai T Haga S Taniguchi N Tajiri H . Beta2-adrenergic receptor regulates Toll-like receptor-4-induced nuclear factor-kappaB activation through beta-arrestin 2. Immunology. (2008) 124:348–56. doi: 10.1111/j.1365-2567.2007.02781.x 18194271 PMC2440829

[13] Anderson P . Post-transcriptional regulons coordinate the initiation and resolution of inflammation. Nat Rev Immunol. (2010) 10:24–35. doi: 10.1038/nri2685 20029446

[14] Bartel DP . MicroRNAs: genomics, biogenesis, mechanism, and function. Cell. (2004) 116:281–97. doi: 10.1016/S0092-8674(04)00045-5 14744438

[15] Bartel DP . Metazoan microRNAs. Cell. (2018) 173:20–51. doi: 10.1016/j.cell.2018.03.006 29570994 PMC6091663

[16] Recchiuti A Krishnamoorthy S Fredman G Chiang N Serhan CN . MicroRNAs in resolution of acute inflammation: identification of novel resolvin D1-miRNA circuits. FASEB J. (2011) 25:544–60. doi: 10.1096/fj.10-169599 20956612 PMC3023392

[17] Rius B Titos E Morán-Salvador E López-Vicario C García-Alonso V González-Périz A . Resolvin D1 primes the resolution process initiated by calorie restriction in obesity-induced steatohepatitis. FASEB J. (2014) 28:836–48. doi: 10.1096/fj.13-235614 24249635

[18] Codagnone M Cianci E Lamolinara A Mari VC Nespoli A Isopi E . Resolvin D1 enhances the resolution of lung inflammation caused by long-term Pseudomonas aeruginosa infection. Mucosal Immunol. (2018) 11:35–49. doi: 10.1038/mi.2017.36 28422188

[19] Li Y Dalli J Chiang N Baron RM Quintana C Serhan CN . Plasticity of leukocytic exudates in resolving acute inflammation is regulated by MicroRNA and proresolving mediators. Immunity. (2013) 39:885–98. doi: 10.1016/j.immuni.2013.10.011 24238341 PMC3888517

[20] Becker LE Lu Z Chen W Xiong W Kong M Li Y . A systematic screen reveals MicroRNA clusters that significantly regulate four major signaling pathways. PloS One. (2012) 7:e48474. doi: 10.1371/journal.pone.0048474 23144891 PMC3493556

[21] da Rocha ST Edwards CA Ito M Ogata T Ferguson-Smith AC . Genomic imprinting at the mammalian Dlk1-Dio3 domain. Trends Genet. (2008) 24:306–16. doi: 10.1016/j.tig.2008.03.011 18471925

[22] Wang Y Mei C Su X Wang H Yang W Zan L . MEF2A regulates the MEG3-DIO3 miRNA mega cluster-targeted PP2A signaling in bovine skeletal myoblast differentiation. Int J Mol Sci. (2019) 20:2748. doi: 10.3390/ijms20112748 31167510 PMC6600538

[23] Farhadova S Ghousein A Charon F Surcis C Gomez-Velazques M Roidor C . The long non-coding RNA Meg3 mediates imprinted gene expression during stem cell differentiation. Nucleic Acids Res. (2024) 52:6183–200. doi: 10.1093/nar/gkae247 38613389 PMC11194098

[24] Weinberg-Shukron A Youngson NA Ferguson-Smith AC Edwards CA . Epigenetic control and genomic imprinting dynamics of the Dlk1-Dio3 domain. Front Cell Dev Biol. (2023) 11:1328806. doi: 10.3389/fcell.2023.1328806 38155837 PMC10754522

[25] Dai R Wang Z Ahmed SA . Epigenetic contribution and genomic imprinting dlk1-dio3 miRNAs in systemic lupus erythematosus. Genes (Basel). (2021) 12:680. doi: 10.3390/genes12050680 34062726 PMC8147206

[26] Gilbert FB Cunha P Jensen K Glass EJ Foucras G Robert-Granié C . Differential response of bovine mammary epithelial cells to Staphylococcus aureus or Escherichia coli agonists of the innate immune system. Vet Res. (2013) 44:40. doi: 10.1186/1297-9716-44-40 23758654 PMC3686618

[27] Zhang L Hou X Sun L He T Wei R Pang M . Staphylococcus aureus bacteriophage suppresses LPS-induced inflammation in MAC-T bovine mammary epithelial cells. Front Microbiol. (2018) 9:1614. doi: 10.3389/fmicb.2018.01614 30083140 PMC6064726

[28] Fusco R Cordaro M Siracusa R Peritore AF D'Amico R Licata P . Effects of hydroxytyrosol against lipopolysaccharide-induced inflammation and oxidative stress in bovine mammary epithelial cells: A natural therapeutic tool for bovine mastitis. Antioxidants (Basel). (2020) 9:693. doi: 10.3390/antiox9080693 32756342 PMC7464001

[29] Robinson MD McCarthy DJ Smyth GK . edgeR: a Bioconductor package for differential expression analysis of digital gene expression data. Bioinformatics. (2010) 26:139–40. doi: 10.1093/bioinformatics/btp616 19910308 PMC2796818

[30] Schmitz Nunes V Rogério AP Abrahão OJ . Insights into the activation mechanism of the ALX/FPR2 receptor. J Phys Chem Lett. (2020) 11:8952–57. doi: 10.1021/acs.jpclett.0c02052 33030905

[31] Nam K Dos Santos HT Maslow FM Davis DJ Galloway TL Dooley LM . Activation of human FPR2 with AT-rvD1 resolves acute sialadenitis *in vivo*. Inflammation. (2025) 48:4189–202. doi: 10.1007/s10753-025-02320-6 40464830 PMC12353947

[32] Liu W Huang J Doycheva D Gamdzyk M Tang J Zhang JH . RvD1 binding with FPR2 attenuates inflammation via Rac1/NOX2 pathway after neonatal hypoxic-ischemic injury in rats. Exp Neurol. (2019) 320:112982. doi: 10.1016/j.expneurol.2019.112982 31247196 PMC6708447

[33] Gabl M Holdfeldt A Sundqvist M Lomei J Dahlgren C Forsman H . FPR2 signaling without β-arrestin recruitment alters the functional repertoire of neutrophils. Biochem Pharmacol. (2017) 145:114–22. doi: 10.1016/j.bcp.2017.08.018 28855087

[34] Zhang S Gong H Ge Y Ye RD . Biased allosteric modulation of formyl peptide receptor 2 leads to distinct receptor conformational states for pro- and anti-inflammatory signaling. Pharmacol Res. (2020) 161:105117. doi: 10.1016/j.phrs.2020.105117 32768626

[35] Krishnamoorthy S Recchiuti A Chiang N Yacoubian S Lee CH Yang R . Resolvin D1 binds human phagocytes with evidence for proresolving receptors. Proc Natl Acad Sci USA. (2010) 107:1660–65. doi: 10.1073/pnas.0907342107 20080636 PMC2824371

[36] Gao H Sun Y Wu Y Luan B Wang Y Qu B . Identification of beta-arrestin2 as a G protein-coupled receptor-stimulated regulator of NF-kappaB pathways. Mol Cell. (2004) 14:303–17. doi: 10.1016/S1097-2765(04)00216-3 15125834

[37] Krishnamoorthy S Recchiuti A Chiang N Fredman G Serhan CN . Resolvin D1 receptor stereoselectivity and regulation of inflammation and proresolving microRNAs. Am J Pathol. (2012) 180:2018–27. doi: 10.1016/j.ajpath.2012.01.028 22449948 PMC3349829

[38] Taganov KD Boldin MP Chang KJ Baltimore D . NF-kappaB-dependent induction of microRNA miR-146, an inhibitor targeted to signaling proteins of innate immune responses. Proc Natl Acad Sci USA. (2006) 103:12481–86. doi: 10.1073/pnas.0605298103 16885212 PMC1567904

[39] Nayak S Aich M Kumar A Sengupta S Bajad P Dhapola P . Novel internal regulators and candidate miRNAs within miR-379/miR-656 miRNA cluster can alter cellular phenotype of human glioblastoma. Sci Rep. (2018) 8:7673. doi: 10.1038/s41598-018-26000-8 29769662 PMC5955984

[40] Thomson JM Newman M Parker JS Morin-Kensicki EM Wright T Hammond SM . Extensive post-transcriptional regulation of microRNAs and its implications for cancer. Genes Dev. (2006) 20:2202–07. doi: 10.1101/gad.1444406 16882971 PMC1553203

[41] Sheedy FJ Palsson-McDermott E Hennessy EJ Martin C O'Leary JJ Ruan Q . Negative regulation of TLR4 via targeting of the proinflammatory tumor suppressor PDCD4 by the microRNA miR-21. Nat Immunol. (2010) 11:141–47. doi: 10.1038/ni.1828 19946272

[42] Takaesu G Kishida S Hiyama A Yamaguchi K Shibuya H Irie K . TAB2, a novel adaptor protein, mediates activation of TAK1 MAPKKK by linking TAK1 to TRAF6 in the IL-1 signal transduction pathway. Mol Cell. (2000) 5:649–58. doi: 10.1016/S1097-2765(00)80244-0 10882101

[43] Moussion C Ortega N Girard JP . The IL-1-like cytokine IL-33 is constitutively expressed in the nucleus of endothelial cells and epithelial cells *in vivo*: a novel 'alarmin'? PloS One. (2008) 3:e3331. doi: 10.1371/journal.pone.0003331 18836528 PMC2556082

[44] Liao Z Dong J Wu W Yang T Wang T Guo L . Resolvin D1 attenuates inflammation in lipopolysaccharide-induced acute lung injury through a process involving the PPARγ/NF-κB pathway. Respir Res. (2012) 13:110. doi: 10.1186/1465-9921-13-110 23199346 PMC3545883

[45] Lee HN Kundu JK Cha YN Surh YJ . Resolvin D1 stimulates efferocytosis through p50/p50-mediated suppression of tumor necrosis factor-α expression. J Cell Sci. (2013) 126:4037–47. doi: 10.1242/jcs.131003 23788426

[46] Zhong H Voll RE Ghosh S . Phosphorylation of NF-kappa B p65 by PKA stimulates transcriptional activity by promoting a novel bivalent interaction with the coactivator CBP/p300. Mol Cell. (1998) 1:661–71. doi: 10.1016/S1097-2765(00)80066-0 9660950

[47] Ngo KA Kishimoto K Davis-Turak J Pimplaskar A Cheng Z Spreafico R . Dissecting the regulatory strategies of NF-κB relA target genes in the inflammatory response reveals differential transactivation logics. Cell Rep. (2020) 30:2758–75. doi: 10.1016/j.celrep.2020.01.108 32101750 PMC7061728

[48] Smale ST . Hierarchies of NF-κB target-gene regulation. Nat Immunol. (2011) 12:689–94. doi: 10.1038/ni.2070 21772277 PMC3169328

[49] Kwon JW Kwon HK Shin HJ Choi YM Anwar MA Choi S . Activating transcription factor 3 represses inflammatory responses by binding to the p65 subunit of NF-κB. Sci Rep. (2015) 5:14470. doi: 10.1038/srep14470 26412238 PMC4585983

[50] Peng Y Xiong RP Wang B Chen X Ning YL Zhao Y . c-Ski is a novel repressor of NF-κB through interaction with p65 and HDAC1 in U937 cells. Cell Commun Signal. (2025) 23:165. doi: 10.1186/s12964-025-02178-z 40176138 PMC11967118

[51] Kang Y Kim YW Kang J Kim A . Histone H3K4me1 and H3K27ac play roles in nucleosome eviction and eRNA transcription, respectively, at enhancers. FASEB J. (2021) 35:e21781. doi: 10.1096/fj.202100488R 34309923

[52] Borghini L Hibberd M Davila S . Changes in H3K27ac following lipopolysaccharide stimulation of nasopharyngeal epithelial cells. BMC Genomics. (2018) 19:969. doi: 10.1186/s12864-018-5295-4 30587130 PMC6307289

[53] Tamura I Maekawa R Jozaki K Ohkawa Y Takagi H Doi-Tanaka Y . Transcription factor C/EBPβ induces genome-wide H3K27ac and upregulates gene expression during decidualization of human endometrial stromal cells. Mol Cell Endocrinol. (2021) 520:111085. doi: 10.1016/j.mce.2020.111085 33232782

[54] Hilton IB D'Ippolito AM Vockley CM Thakore PI Crawford GE Reddy TE . Epigenome editing by a CRISPR-Cas9-based acetyltransferase activates genes from promoters and enhancers. Nat Biotechnol. (2015) 33:510–7. doi: 10.1038/nbt.3199 25849900 PMC4430400

[55] Mottola G Chatterjee A Wu B Chen M Conte MS . Aspirin-triggered resolvin D1 attenuates PDGF-induced vascular smooth muscle cell migration via the cyclic adenosine monophosphate/protein kinase A (cAMP/PKA) pathway. PloS One. (2017) 12:e0174936. doi: 10.1371/journal.pone.0174936 28362840 PMC5376330

[56] Wang Q Zheng X Cheng Y Zhang YL Wen HX Tao Z . Resolvin D1 stimulates alveolar fluid clearance through alveolar epithelial sodium channel, Na,K-ATPase via ALX/cAMP/PI3K pathway in lipopolysaccharide-induced acute lung injury. J Immunol. (2014) 192:3765–77. doi: 10.4049/jimmunol.1302421 24646745

[57] Gu Z Lamont GJ Lamont RJ Uriarte SM Wang H Scott DA . Resolvin D1, resolvin D2 and maresin 1 activate the GSK3β anti-inflammatory axis in TLR4-engaged human monocytes. Innate Immun. (2016) 22:186–95. doi: 10.1177/1753425916628618 26878867 PMC5257244

[58] Mayr B Montminy M . Transcriptional regulation by the phosphorylation-dependent factor CREB. Nat Rev Mol Cell Biol. (2001) 2:599–609. doi: 10.1038/35085068 11483993

