# Supplementary material for: Resolvin D1 engages FPR2–ARRB2 signaling and chromatin–microRNA regulation to restrain inflammatory activation in bovine mammary epithelial cells
Source: Front Immunol. 2026 Oct 23;17:1978720. doi: 10.3389/fimmu.2026.1978720 (PMC13739536; doi:10.3389/fimmu.2026.1978720)
Supplement: Supplementary file 1 [file Supplementaryfile1.docx]

Supplementary Figure S1. Validation of LPS-responsive TLR4 signaling in MAC-T cells.
(A) Western blot analysis of TLR4 after LPS stimulation for 1 h.
(B–C) Western blot analysis and quantification of p-IRF3/IRF3 after LPS stimulation for 0, 15, 30, 60, or 120 min.
(D–E) Cells were stimulated with LPS for 0, 15, 30, 60, or 120 min. Lysates were immunoprecipitated with an anti-TRAM antibody and immunoblotted for TRIF. IgG served as the negative control. Co-immunoprecipitated TRIF was normalized to immunoprecipitated TRAM.
Data are mean ± SEM from three independent experiments. **P < 0.01.

Supplementary Figure S2. Validation of gene knockdown, miRNA modulation, predicted miRNA-binding sites, and p65–HDAC interactions.
(A) Western blot screening of three ARRB2-targeting siRNAs after transfection with siRNA1, siRNA2, siRNA3, or siNC for 48 h.

(B–C) RT-qPCR analysis of mature bta-miR-380-5p and bta-miR-654 after transfection with the corresponding mimic or mimic NC for 48 h.
(D–F) Western blot validation of p65 (D), HDAC1 (E), and CREB1 (F) knockdown in untransfected control cells or cells transfected with siNC or the corresponding target siRNA for 48 h.
(G) Predicted binding sites and mutant sequences for bta-miR-380-5p in the TAB2 3′ UTR and bta-miR-654 in the IL-33 3′ UTR. Red sequences indicate the miRNA seed-matched sites and corresponding mutant sequences.
(H) STRING-predicted protein interactions of RELA/p65 with HDAC1, HDAC2, and HDAC3 (CC BY 4.0). Data are presented as the mean ± SEM; statistical analyses were performed as described in the Materials and Methods. ns, not significant; *P < 0.05; **P < 0.01.

Supplementary Figure S3. Representative Western blot images containing molecular-weight marker lanes.

The following images correspond to Western blot experiments shown in Figures 1, 2, 3, 5, 6, 7, 8, and 9 and Supplementary Figure S1.

Supplementary Table S1A. Predicted RELA/p65-binding sites within the bovine MEG3 promoter-proximal region

| **Predicted site** | **Predicted binding sequence (5′–3′)** | **Position (Relative to TSS)** | **Raw Score** | **Relative score** | **Strand** |
| --- | --- | --- | --- | --- | --- |
| p65-1 | CGGACTTTCG | −258 to −249 bp | 8.403238 | 0.8203250 | ＋ |
| p65-2 | TGGAATTGCC | −41 to −32 bp | 9.691203 | 0.8540289 | − |
| p65-3 | GGGGTTGTCC | +257 to +266 bp | 8.096203 | 0.8122904 | − |

Binding sites were predicted using the JASPAR CORE vertebrate RELA matrix MA0107.1. Coordinates are shown relative to the annotated MEG3 transcription start site (+1). The “+” and “−” symbols indicate the forward and reverse strands, respectively. Sites with a relative score of ≥0.80 were retained.

Supplementary Table S1B. Predicted CREB1-binding sites within the bovine MEG3 promoter-proximal region

| **Predicted site** | **Predicted binding sequence (5′–3′)** | **Position (Relative to TSS)** | **Raw Score** | **Relative score** | **Strand** |
| --- | --- | --- | --- | --- | --- |
| CREB1-1 | TGAAGACA | −748 to −741 bp | 7.046975 | 0.8285390 | − |
| CREB1-2 | TCAGGTCA | +323 to +330 bp | 6.544801 | 0.8171910 | − |
| CREB1-3 | TGTGGTCA | +351 to +358 bp | 6.514432 | 0.8165047 | + |

Binding sites were predicted using the JASPAR CORE vertebrate CREB1 matrix MA0018.5. Coordinates are shown relative to the annotated MEG3 transcription start site (+1). The “+” and “−” symbols indicate the forward and reverse strands, respectively. Sites with a relative score of ≥0.80 were retained.

Supplementary Table S2A. Stem-loop RT primer sequences used for miRNA reverse transcription

| **Target** | **Stem-loop RT primer sequence (5′–3′)** |
| --- | --- |
| bta-miR-16a | GTCGTATCCAGTGCAGGGTCCGAGGTATTCGCACTGGATACGACCACCAA |
| bta-miR-380-5p | GTCGTATCCAGTGCAGGGTCCGAGGTATTCGCACTGGATACGACGCGCAT |
| bta-miR-370 | GTCGTATCCAGTGCAGGGTCCGAGGTATTCGCACTGGATACGACACCAGG |
| bta-miR-654 | GTCGTATCCAGTGCAGGGTCCGAGGTATTCGCACTGGATACGACAAGGTG |
| bta-miR-381 | GTCGTATCCAGTGCAGGGTCCGAGGTATTCGCACTGGATACGACACAGAG |
| bta-miR-411a | GTCGTATCCAGTGCAGGGTCCGAGGTATTCGCACTGGATACGACCGTACG |
| bta-miR-432 | GTCGTATCCAGTGCAGGGTCCGAGGTATTCGCACTGGATACGACCCACCC |

Supplementary Table S2B. Primer sequences used for mRNA and lncRNA qPCR analysis

| **Target** | **Forward primer (5′–3′)** | **Reverse primer (5′–3′)** |
| --- | --- | --- |
| IL-6 | ATGATGAGTGTGAAAGCAGCAAGG | TGATACTCCAGAAGACCAGCAGTG |
| IL-8 | GCTGGCTGTTGCTCTCTTGG | GGGTGGAAAGGTGTGGAATGTG |
| MEG3 | TAATCTTCGCTTGCCTCCCC | TAAGCGTCCCAGTCCCAAAC |
| GAPDH | TCACCACCATGGAGAAGGC | GCTAAGCAGTTGGTGGTGCA |

Supplementary Table S2C. Primer sequences used for miRNA qPCR analysis

| **Target** | **Forward primer (5′–3′)** | **Reverse primer (5′–3′)** |
| --- | --- | --- |
| bta-miR-16a | GCGCTAGCAGCACGTAAATA | ATCCAGTGCAGGGTCCGAGG |
| bta-miR-380-5p | GCGCTGGTTGACCATAGAAC | ATCCAGTGCAGGGTCCGAGG |
| bta-miR-370 | GCCTGCTGGGGTGGAA | ATCCAGTGCAGGGTCCGAGG |
| bta-miR-654 | CGCTATGTCTGCTGACCAT | ATCCAGTGCAGGGTCCGAGG |
| bta-miR-381 | CGCTATACAAGGGCAAGCT | ATCCAGTGCAGGGTCCGAGG |
| bta-miR-411a | CGCGCATAGTAGACCGTATAG | ATCCAGTGCAGGGTCCGAGG |
| bta-miR-432 | GCGCTCTTGGAGTAGGTCATT | ATCCAGTGCAGGGTCCGAGG |

Supplementary Table S2D. Primers used for CUT&Tag-qPCR

| **Target region** | **Primer** | **Sequence (5′–3′)** | **Amplicon size (bp)** |
| --- | --- | --- | --- |
| p65-P1 | Forward | ACTTCTCAGGAGGGAGACCTGGG | 120 |
| p65-P1 | Reverse | CCGGTACGATGACTTGACGGTCC |  |
| p65-P2 | Forward | GGCTAAGTGTTGAAACCAGTGCCC | 142 |
| p65-P2 | Reverse | AGTTGGACCTCCAAAGTGGTAGGG |  |
| p65-P3 | Forward | CCTCACCTGTCTCACGCTTCTCG | 129 |
| p65-P3 | Reverse | CCCGACTGGACTCAGTCCTGTAC |  |
| CREB1-P1 | Forward | AGTGCCGGCCGTTAGAGGTCATC | 125 |
| CREB1-P1 | Reverse | GGTGGTTCCTGGTGGACACCTAC |  |
| CREB1-P2 | Forward | GCTCTCGGTGTACCAGGTAGGG | 150 |
| CREB1-P2 | Reverse | GTCTACAGGTACACTCGCTCCGG |  |
